# Microfluidic platform for the reproduction of hypoxic vascular microenvironments

**DOI:** 10.1038/s41598-023-32334-9

**Published:** 2023-04-03

**Authors:** Naoyuki Takahashi, Daisuke Yoshino, Ryuji Sugahara, Satomi Hirose, Kazuki Sone, Jean-Paul Rieu, Kenichi Funamoto

**Affiliations:** 1 Graduate School of Biomedical Engineering, Tohoku University, 6-6-12 Aramaki-aza Aoba, Aoba-ku, Sendai, Miyagi 980-8579 Japan; 2 Institute of Fluid Science, Tohoku University, 2-1-1 Katahira, Aoba-ku, Sendai, Miyagi 980-8577 Japan; 3 Institute of Engineering, Tokyo University of Agriculture and Technology, 2-24-16 Naka-cho, Koganei, Tokyo 184-8588 Japan; 4 Institut Lumière Matière, UMR5306, Université Lyon 1-CNRS, Université de Lyon, 69622 Villeurbanne, France; 5 Graduate School of Engineering, Tohoku University, 6-6-1 Aramaki-aza Aoba, Aoba-ku, Sendai, Miyagi 980-8597 Japan

**Keywords:** Collective cell migration, Biomedical engineering

## Abstract

Vascular endothelial cells (ECs) respond to mechanical stimuli caused by blood flow to maintain vascular homeostasis. Although the oxygen level in vascular microenvironment is lower than the atmospheric one, the cellular dynamics of ECs under hypoxic and flow exposure are not fully understood. Here, we describe a microfluidic platform for the reproduction hypoxic vascular microenvironments. Simultaneous application of hypoxic stress and fluid shear stress to the cultured cells was achieved by integrating a microfluidic device and a flow channel that adjusted the initial oxygen concentration in a cell culture medium. An EC monolayer was then formed on the media channel in the device, and the ECs were observed after exposure to hypoxic and flow conditions. The migration velocity of the ECs immediately increased after flow exposure, especially in the direction opposite to the flow direction, and gradually decreased, resulting in the lowest value under the hypoxic and flow exposure condition. The ECs after 6-h simultaneous exposure to hypoxic stress and fluid shear stress were generally aligned and elongated in the flow direction, with enhanced VE-cadherin expression and actin filament assembly. Thus, the developed microfluidic platform is useful for investigating the dynamics of ECs in vascular microenvironments.

## Introduction

Blood vessels play important roles in a wide variety of in vivo phenomena such as homeostasis, development, organ formation, and pathological processes. The blood vessel lumen is covered with a monolayer of vascular endothelial cells (ECs), which are deeply involved in the process of exchanging nutrients and waste products between blood and tissues. ECs are continuously exposed to mechanical stimuli caused by blood flow^1^, such as fluid shear stress of up to several Pa, and change their morphology and characteristics to maintain vascular function^2^. For instance, ECs orientate and elongate in the blood flow direction, sensing the fluid shear stress^3^ and assembling actin stress fibers, which increases their mechanical stiffness^4^. Also, ECs show collective cell migration using a mechanism different from single cell migration^5,6^, affecting adjacent cells and forming multiple clusters^7–9^. This migratory behavior is closely related to physiological and pathological events, including morphogenesis^10,11^, tissue regeneration, and tumor progression^12^. Collective cell migration varies with different stimuli owing to intracellular signal transduction which controls cell-substrate^13^, cell-extracellular matrix (ECM)^10,11^, and cell–cell adhesion molecules, especially vascular endothelial (VE)-cadherin^14,15^, and cytoskeleton remodeling^16^. Moreover, an in vivo oxygen concentration is lower than that in the atmosphere of 21% O_2_. Even in blood flowing in blood vessels, the oxygen concentration is reportedly between 13% (for arterial blood) and 5% (for venous blood), which are considered as physiological normoxia^17^. It is noted that conventional cellular experiments had usually been performed in 21% O_2_, not addressing the physiologically normoxic condition. The oxygen concentration can be further decreased to be severe hypoxia in organs and tissues by disease and inflammation^18^, and such hypoxic conditions could change the cellular dynamics of ECs^19–21^. However, the effects of a hypoxic vascular environment on the collective migration of ECs are not fully understood.

Of the observation methods used to study cellular dynamics, cellular experiments with microfluidic devices are particularly promising and extensively performed^22^. Microfluidic devices containing micro-size channels for bioanalysis and chemical reactions enable real-time, high-resolution observation of cells cultured in the channels, and allow the control of multiple environmental factors^1,23^. For example, the precise control of oxygen concentration in cellular experiments using microfluidic devices has been investigated^24,25^. We developed microfluidic devices to reproduce in vivo hypoxic microenvironments^26–28^, and investigated the responses of an EC monolayer formed in the device to hypoxic exposure^29,30^. A confluent EC monolayer exposed to a hypoxic stress of around 3% O_2_ exhibited increased permeability and lost a size-selective barrier function^31^. In addition, collective migration of ECs measured by particle image velocimetry (PIV) using a time-series of phase-contrast microscopic images revealed an oxygen-dependent variation in migration speed, which increased at around 3% O_2_ but decreased at < 1% O_2_^29^. Although our previous experiments on vascular endothelial responses to hypoxia were performed under a static condition without flow exposure for simplification, ECs in an in vivo vascular microenvironment are exposed to blood flows. Few microfluidic devices can simultaneously reproduce shear stress by blood flow and the hypoxic microenvironment observed in vivo^32^ and thus the effects of complex stimuli on the cellular dynamics of a confluent monolayer of ECs remain unclear.

**Figure 1:**
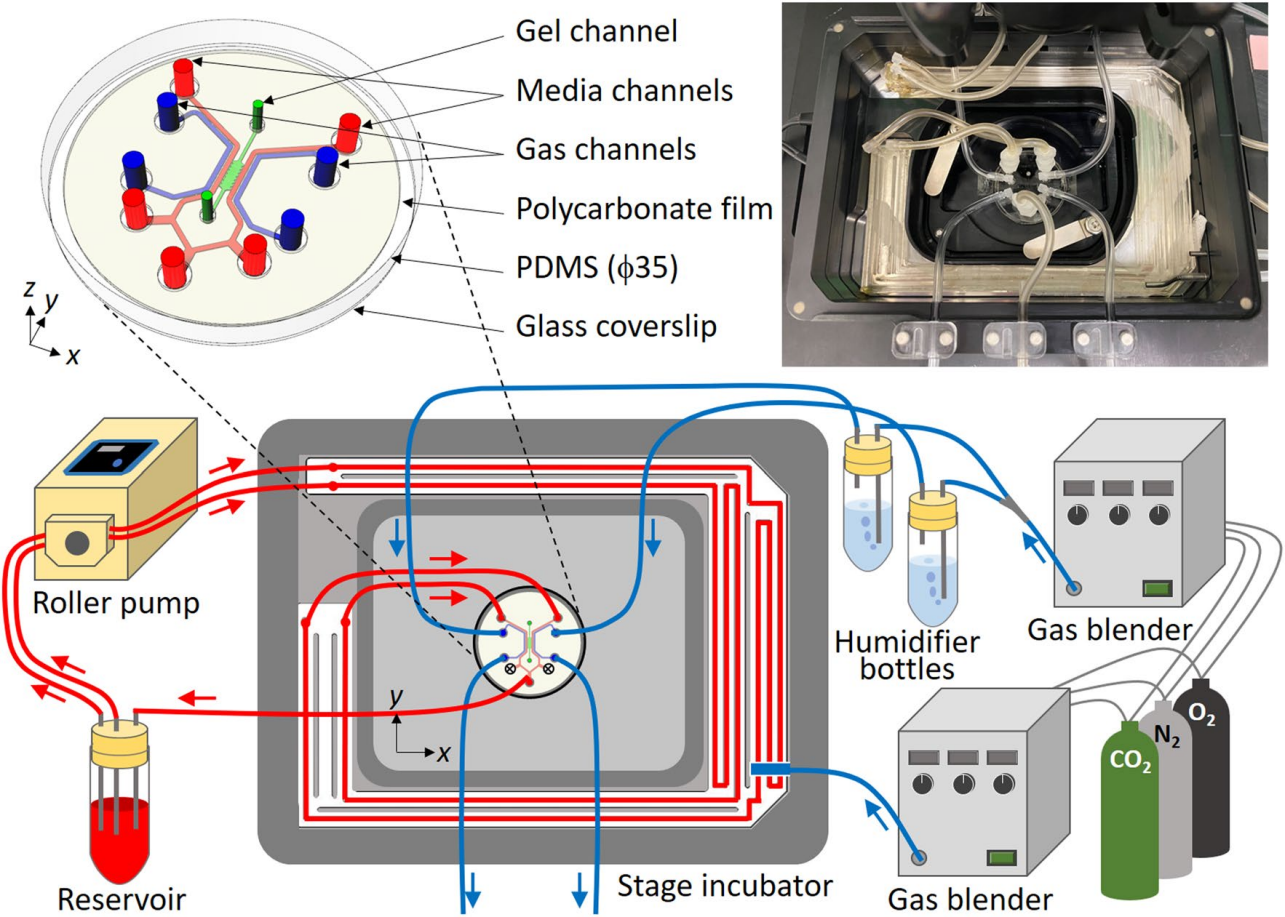
The experimental platform, consisting of a microfluidic device, a flow channel placed in a stage incubator to control the oxygen concentration in a cell culture medium, a roller pump, a reservoir for the cell culture medium, gas blenders, and humidifier bottles. The oxygen and carbon dioxide concentrations of the gas mixtures were adjusted by mixing with nitrogen. The gas mixtures were humidified by bubbling through the humidifier bottles before being supplied to the gas channels in the microfluidic device. The cell culture medium was circulated by the roller pump: medium was drawn from the reservoir, supplied to the flow channels and the media channels in the microfluidic device, and then returned to the reservoir. Red and blue arrows indicate the flow directions of the cell culture medium and gas mixtures, respectively. The upper right figure is a photograph of the device and the flow channel placed in the stage incubator.

In this study, we developed a microfluidic platform allowing the application of hypoxic stress and fluid shear stress to ECs (Fig. 1 and Supplementary Fig. S1). Based on three-dimensional (3D) numerical simulation, experimental methods to generate hypoxic and flow exposure conditions to ECs were considered, and a flow channel for oxygen control in a cell culture medium was fabricated and integrated with a microfluidic device. Oxygen concentrations generated inside the device were experimentally validated with an oxygen-sensing film. The feasibility of the platform was then examined by investigating the collective migration and morphological changes of ECs. The migration of ECs formed a monolayer in the device were measured while controlling the oxygen concentration at a normoxic 21% O_2_ or a hypoxic < 5% O_2_; subsequently, cell morphology and cell–cell adhesion were evaluated by microscopic observation of the ECs with immunofluorescence staining. The results indicate that flow exposure increases the collective migration of ECs at the early stage of exposure to fluid flow and hypoxic exposure promotes their orientation to the flow direction.

## Results

### Optimization of experimental conditions by computational analysis

Computational analysis was used to identify experimental conditions for the simultaneous application of hypoxic stress and fluid shear stress to the ECs cultured on the media channel. In the absence of media flow (*Pe*_m_ = 0), the condition H0_stat_ that supplied an anoxic gas mixture (*c*_g_ = 0%) only to the gas channels decreased oxygen concentration in the device as the flowrate or Péclet number *Pe*_g_ of the gas mixture increased. However, at *Pe*_g_ ≥ 100, the oxygen concentration remained essentially unchanged in the condition H0_stat_^26^. To decrease the amount of gas mixture used and decrease the pressure generated in the gas channels, the flowrate of the gas mixture was set to 18 ml/min (*Pe*_g_ = 100). In the conditions H0_stat_-H10_stat_ that supplied a gas mixture to the gas channels at *c*_g_ = 0, 1, 3, 5, and 10%, the oxygen concentration at the centerline on the bottom of the media channel was 0.8, 1.7, 3.7, 5.6, and 10.4%, respectively (Fig. S2). This indicates that the oxygen concentration in the device was approximately 1% higher than that in the supplied gas mixture due to oxygen infusion from the surrounding atmosphere. However, there was a linear relationship between the oxygen concentrations in the gas and media channels, so the oxygen concentration around the cells could be controlled.

**Figure 2:**
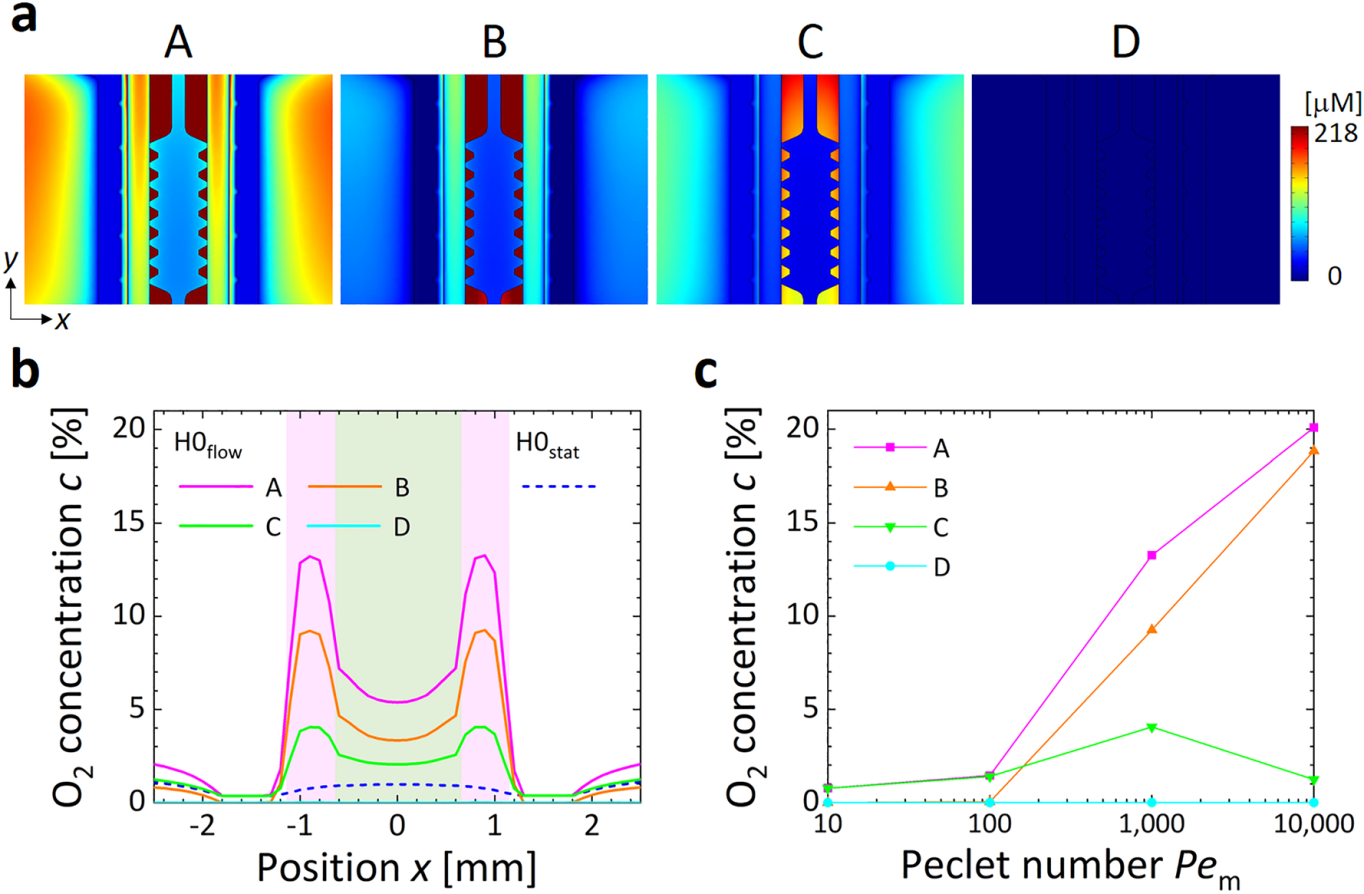
Computational results of steady oxygen concentration in the microfluidic device. (**a**) Oxygen distributions on the horizontal cross section (*z* = 0 mm) and (**b**) profiles of oxygen concentration *c* across the media and gel channels (*y* = 0 mm) under the hypoxic condition H0_flow_ at *Pe*_m_ = 1,000, generated by different oxygen control methods A–D (see Table 3), and for the static condition H0_stat_ at *Pe*_m_ = 0 by the method A. For the method A, a gas mixture without oxygen (*c*_g_ = 0%) was supplied only to both gas channels. For the methods B, C, and D, the oxygen concentrations either in the stage incubator *c*_i_, in the supplied medium *c*_m_, or both, was additionally controlled at 0% O_2_. The origin was set at the center of the gel channel, and the *x*-direction was defined as the horizontal direction normal to the gel channel. Regions shaded with pink and green indicate sections of the media and gel channels, respectively. (**c**) Variations in oxygen concentration *c* at the center of the media channel (|*x*|= 0.9 mm, *y* = 0 mm, *z* = 0 mm) with Péclet numbers *Pe*_m_ for the medium flow.

In the media flow (*Pe*_m_ > 0) cases such as the condition H0_flow_ (Fig. 2), it was difficult to maintain a hypoxic state only with the gas supply to the gas channels (the method A). This difficulty was due to convection being dominant rather than diffusion, resulting in the cell culture medium, which had been kept in a normoxic condition (*c*_m_ = 21%), flowing downstream with little exchange of dissolved gas components with the supplied gas mixture (Fig. 2a). The method B, which filled the stage incubator with anoxic gas (*c*_i_ = 0%), generated a low oxygen concentration in the device when *Pe*_m_ ≤ 100 (Fig. 2c). However, this hypoxic condition could not be maintained when *Pe*_m_ was further increased (Fig. 2b). Moreover, the method C, which supplied cell culture medium preconditioned at *c*_m_ = 0% to the media channel in the device placed in a normoxic atmosphere at *c*_i_ = 21%, generated the same oxygen level as the method A when *Pe*_m_ < 100, namely, 0.8% O_2_ (Fig. 2c). However, the oxygen concentration varied when the Péclet number *Pe*_m_ was changed: the oxygen concentration increased to 1.4 and 4.1% O_2_ when *Pe*_m_ = 100 and 1,000, respectively, whereas it decreased to 1.2% O_2_ when *Pe*_m_ = 10,000 (Fig. 2b,c). This variation in oxygen concentration with *Pe*_m_ may be due to the balance between convection and diffusion of oxygen. In contrast, the method D, which simultaneously adjusted the oxygen concentrations *c*_g_, *c*_i_, and *c*_m_, kept the oxygen concentration at the low level. Consequently, because the media flow significantly alters oxygen transportation inside the device, simultaneous control of the oxygen concentrations in the supplied gas mixtures to the gas channels and the stage incubator and preconditioning of the oxygen concentration in a cell culture medium are essential for a flow exposure experiment under a controlled oxygen concentration.

Fluid shear stress τ generated on the bottom of the media channel was essentially uniform in the vicinity of the centerline and proportional to the Péclet number *Pe*_m_ (Supplementary Fig. S3). Assuming that the viscosity and density of the cell culture medium were 1.0 × 10^−3^ Pa s and 1.0 × 10^3^ kg/m^3^, respectively, in the numerical simulation, a flowrate of *Q*_m_ = 100 µl/min (*Pe*_m_ = 5,555) was necessary to generate a fluid shear stress τ of 1 Pa on the bottom of the media channel. In the following cellular experiments, the above flowrate provided a fluid shear stress τ of 0.72 Pa according to the viscosity and density of the cell culture medium measured at 37 °C.

### Validation of the controllability of the oxygen concentration

**Figure 3:**
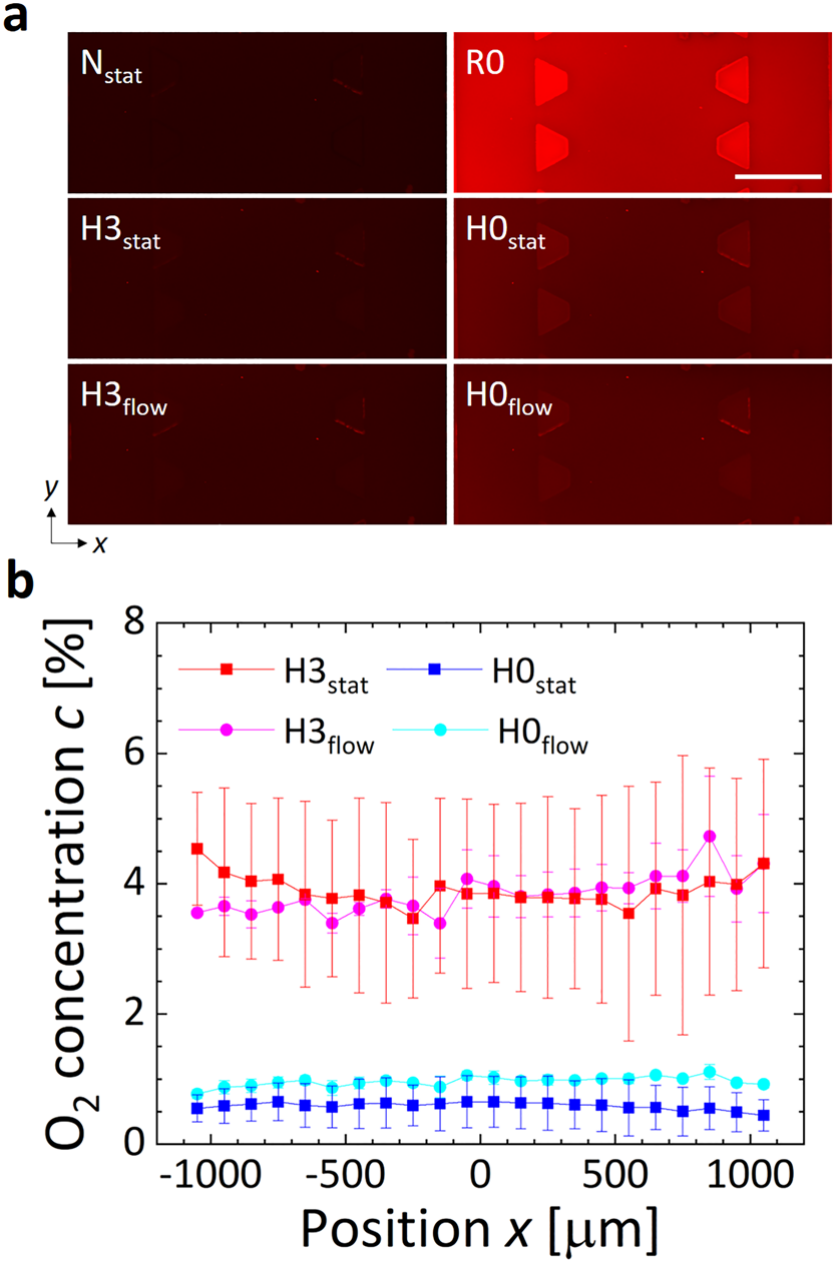
Measurement results of steady oxygen concentration in the microfluidic device. (**a**) Representative microscopic images of phosphorescence from the oxygen-sensing film on the bottom glass cover slip and (**b**) profiles of steady oxygen concentration *c* across the media and gel channels. Under the conditions N_stat_ and R0, the same gas mixtures containing 21 and 0% O_2_ were supplied to the gas channels and the stage incubator, respectively, without media flow. In the hypoxic H3_stat_ and H0_stat_ conditions, gas mixtures containing 3 and 0% O_2_ were supplied only to the gas channels without media flow, respectively, while in the hypoxic H3_flow_ and H0_flow_ conditions, the same gas mixture was supplied to the gas channels and the stage incubator with media flow at *Q*_m_ = 100 µl/min. Scale bar shows 500 μm. Error bars show the standard deviation.

The oxygen concentration in the device was measured by converting the phosphorescence from the oxygen-sensing film (Fig. 3). The raw phosphorescence intensity of PtTFPP in the film varied with the oxygen condition, increasing under low oxygen conditions (Fig. 3a). These microscopic images were used to calculate the oxygen concentrations profiles. The oxygen concentration profiles across the channels under the conditions H3_stat_ and H0_stat_, in which gas mixtures containing 3 and 0% O_2_ were supplied only to the gas channels without media flow, indicated 4.1 and 0.5% O_2_ in the media channel, respectively (Fig. 3b). On the other hand, under the conditions H3_flow_ and H0_flow_, in which gas mixtures containing 3 and 0% O_2_ were supplied to the gas channels and the stage incubator with media flow at *Q*_m_ = 100 µl/min, the oxygen concentration in the media channel was 3.9 and 0.9% O_2_, respectively. The oxygen concentration profiles obtained by measurement and numerical simulation showed good agreement, although there were slight deviations (Figs. S2 and 3b). These results demonstrate the validity of the computational analysis and the controllability of oxygen concentration by the developed microfluidic platform. The simultaneous control of the oxygen concentrations in the supplied gas mixtures to the gas channels and the stage incubator, and preconditioning of the oxygen concentration in a cell culture medium yield a flow exposure experiment under a controlled oxygen concentration.

### Collective cell migration under hypoxic and flow exposure conditions

**Figure 4:**
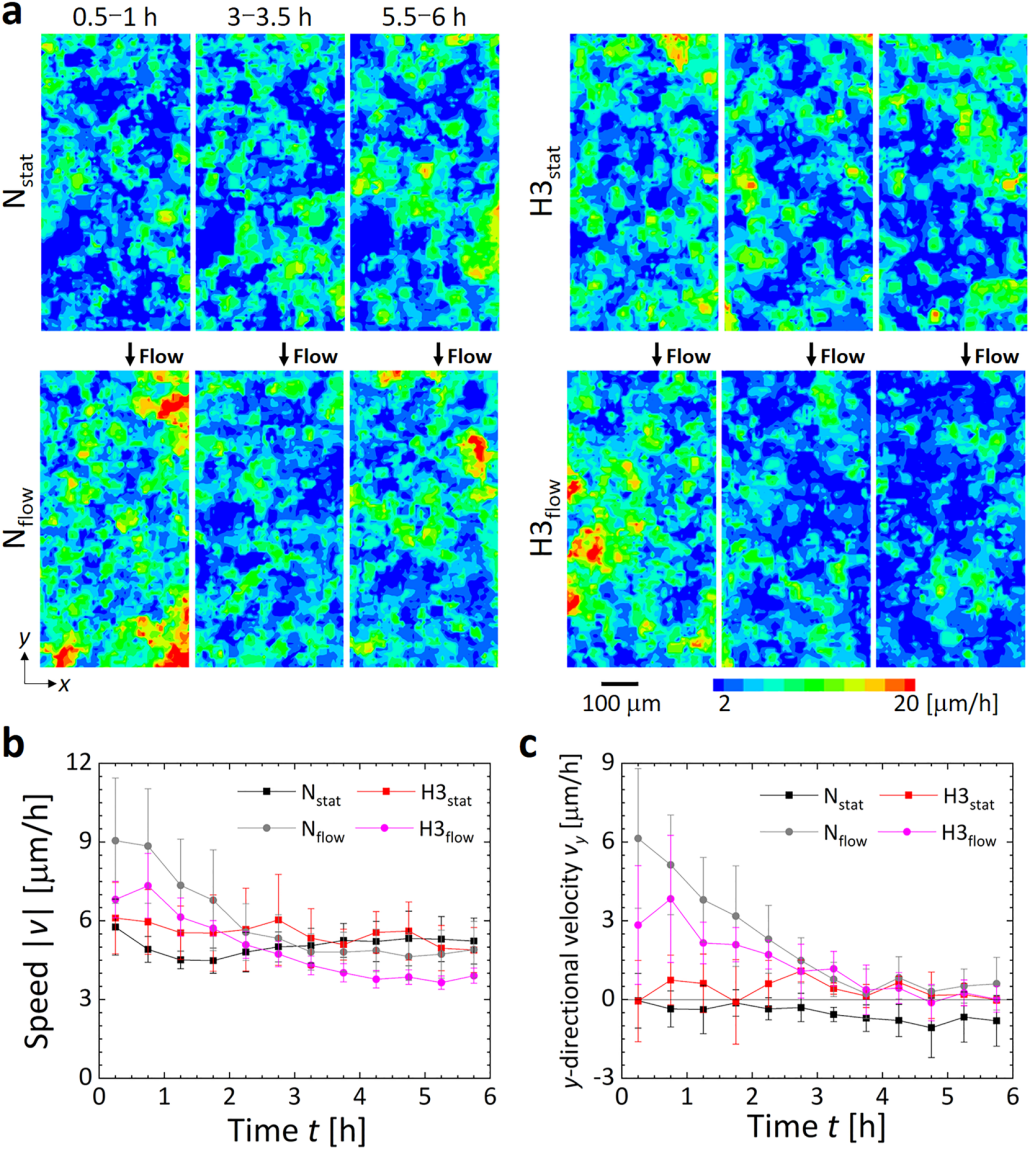
Collective migration of ECs in the monolayer under the four different oxygen and flow conditions N_stat_, H3_stat_, N_flow_, and H3_flow_. When the flow is present, it is applied in the -*y*-direction. (**a**) Contour maps of migration speed |*v*| obtained by PIV analysis using sequential phase-contrast microscopic images at 30 min intervals. Velocity vectors are shown in supplemental materials (Supplementary Figs. S4–S7). (**b**) Time variations of the spatially averaged migration speed |*v*| and the *y*-directional velocity *v*_*y*_ of ECs during a 6-h measurement period while exposing the cells to the oxygen and flow conditions. Error bars show the standard deviation.

Collective migration of ECs was observed on the bottom surface of the media channel under each condition (Supplementary Movie S1). The migration speed of ECs obtained under each condition showed a random distribution of regions with high and low values on the EC monolayer (Fig. 4a). Compared to the normoxic condition N_stat_, regions with relatively high migration speeds were observed slightly more over the entire period under the hypoxic condition H3_stat_ (Supplementary Figs. S4 and S5). Under the conditions with flow exposure (i.e., N_flow_ and H3_flow_), regions with high migration speeds were widely distributed in the early stage of the experiment (< 3 h) (Fig. 4a). The velocity vectors were dominantly in the *y*-direction, which was opposite to the flow direction (Supplementary Figs. S6 and S7). Variations in the space-averaged migration speed of cells without flow exposure were essentially constant throughout the experiment, with higher speeds under the hypoxic condition H3_stat_ than under the normoxic condition N_stat_ from the beginning to the middle of the experiment (Fig. 4b). In contrast, the variation in migration speed of cells under the flow exposure condition was remarkably dependent on the oxygen concentration, showing a maximum value within 1 h after the start of the experiment. The migration speed gradually decreased, with each value becoming lower than that without flow exposure after 4 h. Under the conditions N_flow_ and H3_flow_, the migration speed at 5.5–6 h (just before the end of the experiment) was 0.54- and 0.57-fold of the speed at 0.5–1 h after the start of the experiment, respectively (Supplementary Fig. S8). The migration direction of the cells was evaluated by decomposing the migration velocity into the *x* and *y*-directional components, *v*_*x*_ and *v*_*y*_ (Supplementary Figs. S9a and 4c). ECs under flow exposure migrated in the *y*-direction (i.e., opposite to the flow direction), especially in the early stage of flow exposure. The ratio of the absolute values of the velocity components, |*v*_*y*_|/|*v*_*x*_|, was generally larger than 1 in all conditions, indicating a tendency to migrate relatively easily along the media channel (Supplementary Fig. S9b). Spatial extent of cells migrating in the same direction was evaluated by the spatial autocorrelation function *C*_*vv*_ of the velocity fluctuation vectors *δ***v** obtained by subtracting the spatial average velocity $\overline{\mathbf{v} }$ from the velocity vector **v**:

$${C}_{vv}=\langle \frac{{\sum }_{i}\delta \mathbf{v}\left({\mathbf{r}}_{i}\right)\cdot \delta \mathbf{v}\left({\mathbf{r}}_{i}+\mathbf{r}\right)}{{\sum }_{i}\delta \mathbf{v}\left({\mathbf{r}}_{i}\right)\cdot \delta \mathbf{v}\left({\mathbf{r}}_{i}\right)}\rangle ,$$

where **r**_*i*_ is the positions where velocity vector of cell migration was measured, and the angle brackets denote an average over all directions and time^30,33^. An exponential decrease of the autocorrelation function was observed under each condition (Supplementary Fig. S10). The condition H3_flow_ presented the quickest decay of the autocorrelation function, indicating the cluster sizes of the migrating ECs was the smallest.

### Morphological changes by hypoxic and flow exposure

**Figure 5:**
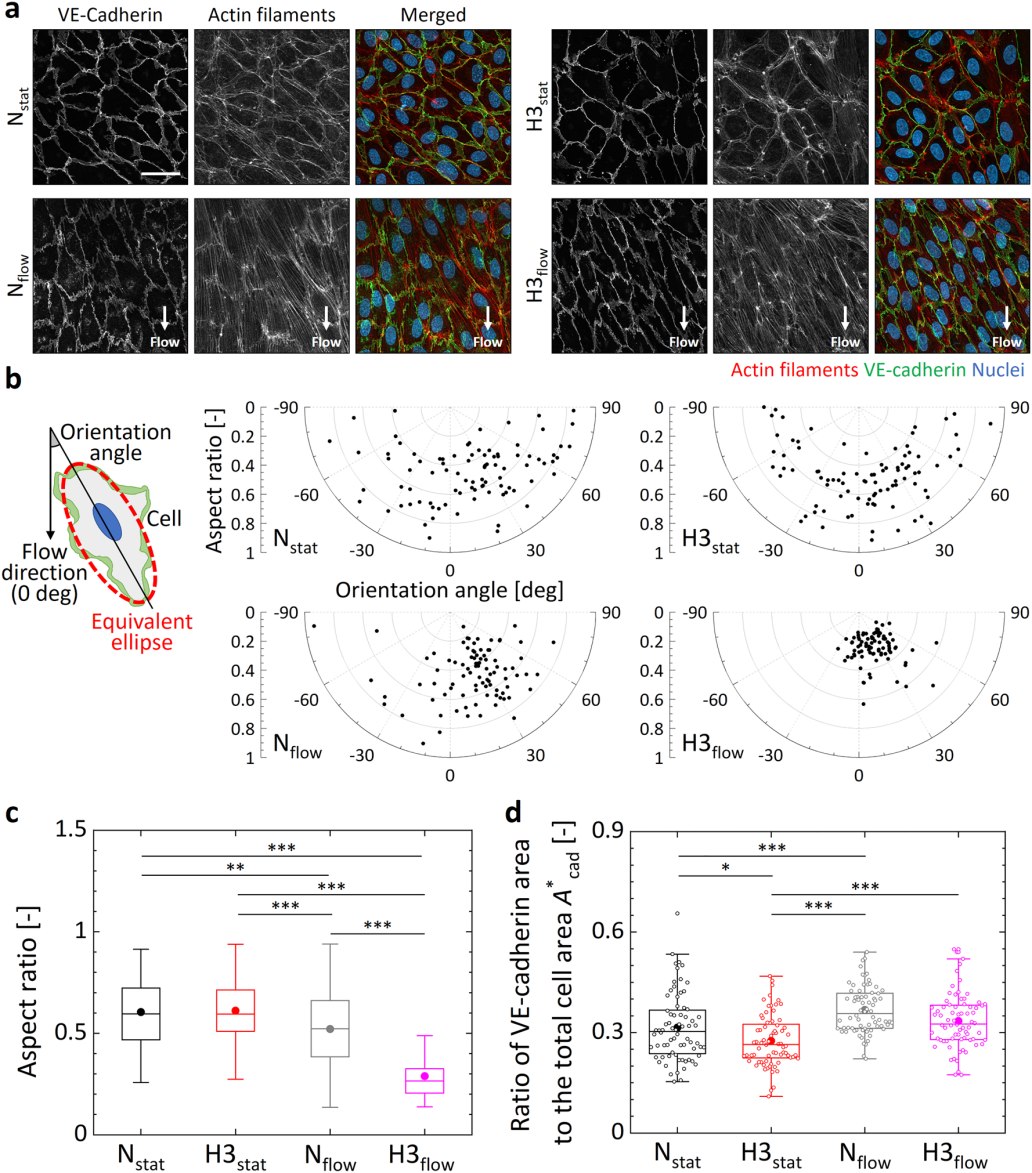
Morphological changes of ECs in the monolayer after 6 h of exposure to the four different oxygen and flow conditions N_stat_, H3_stat_, N_flow_, and H3_flow_. (**a**) Representative images of maximum intensity projections of confocal microscope images of ECs to the *xy*-plane. Scale bar shows 40 μm. (**b**) Orientation angle and aspect ratio of 90 cells from three devices. (**c**) Box-and-whisker plots of the aspect ratio of the cells. The upper and lower extremes represent the maximum and minimum values, the box plot represents quartiles, and the band and dot inside each box show the median and average value, respectively. (**d**) Box-and-whisker plots of the ratio A^*^_cad_ of the VE-cadherin area to the total cell area with the raw data plotted. The metric was measured with 72 cells from three devices for each condition. Significant differences in the metrics at different oxygen and flow conditions were assessed by 2-way ANOVA followed by Tukey’s post-hoc tests for multiple comparisons. *P < 0.05; **P < 0.01; ***P < 0.001.

Microscopic observation by immunofluorescence staining of the ECs showed cell orientation toward the flow direction (Fig. 5a). Cell morphology was evaluated by the orientation angle and aspect ratio of the elliptically approximated cells (Fig. 5b). Regardless of the oxygen condition, the orientation angles and aspect ratios of the cells were distributed over a wide range with various values under no flow exposure conditions. In contrast, their distribution tended to be narrower under flow exposure conditions. Kolmogorov–Smirnov test results for the orientation angles of the cells indicated a significant difference only in the hypoxic condition with flow exposure compared to the normoxic condition without flow exposure (*P* = 0.036). The average value of the aspect ratio and the kurtosis of its distribution were 0.60 and −0.96 under the normoxic condition N_stat_, and 0.61 and −0.26 under the hypoxic condition H3_stat_ (Fig. 5c). On the other hand, the corresponding values were 0.52 and −0.55 in the normoxic condition N_flow_ and 0.29 and 2.37 in the hypoxic condition H3_flow_, indicating that the deviation of the aspect ratio became smaller under flow exposure conditions. Quantification of the orientation and coherency of actin filaments in ECs showed a similar tendency (Fig. S11). The fiber orientation was distributed over a wide range with various values under no flow exposure conditions, while it tended to be narrower under flow exposure conditions. In addition, the coherency was increased by the flow exposure, and showed a maximum value under the hypoxic and flow exposure condition. Thus, the ECs oriented and elongated toward the flow direction by flow exposure, and this tendency was more pronounced under hypoxic conditions.

The area of VE-cadherin acting as intercellular junctions decreased noticeably upon hypoxic exposure, showing narrow and discontinuous bands of VE-cadherin under the condition H3_stat_ (Fig. 5a). In addition, stress fibers developed inside the cells upon flow exposure in the conditions N_flow_ and H3_flow_. Quantitative evaluation of the ratio A^*^_cad_ of the VE-cadherin area to the total cell area showed that the relative area of VE-cadherin was decreased by hypoxic exposure but was increased by flow exposure (Fig. 5d). The relative area of VE-cadherin was largest after 6-h exposure to the normoxic and flow condition, although the migration speed of the cells was highest just after the start of the experiment. The ICAM-1 expression of ECs was increased by the flow exposure, which agreed with the previous report^34^, while it showed large variations by the hypoxic exposure (Fig. S12). These results indicate the phenotype of ECs were also changed by hypoxic and/or flow exposures.

## Discussion

A microfluidic platform was established to circulate cell culture medium while controlling the oxygen concentration, thereby simultaneously applying hypoxic stress and fluid shear stress to a monolayer of ECs. Simultaneous exposure of ECs to hypoxic stress and fluid shear stress was achieved by gas exchange and preconditioning of the oxygen concentration in a cell culture medium. Regardless of the oxygen concentration, the collective migration of ECs against the flow direction immediately increased in the early stage of flow exposure, followed by a decrease to a speed that is slower than that without flow exposure. These results show that morphological changes in the orientation and elongation of ECs caused by flow exposure are promoted when combined with hypoxic exposure.

Based on computational analysis and validation experiments using oxygen sensing-films, we adopted the method to supply gas mixtures to the gas channels at a predefined oxygen concentration and adjusted the oxygen concentrations in the atmosphere around the device and in the cell culture medium to the same level (Figs. 2 and 3). The migration speed of ECs under flow exposure increased immediately after the start of the experiment regardless of the oxygen condition, and then decreased to a speed lower than that observed without flow exposure (Fig. 4b). The increased collective migration by low exposure was predominantly in the *y*-direction, against the flow direction (Fig. 4c and Supplementary Fig. S9). The upstream migration of confluent ECs against fluid shear stress agrees with previous studies^35,36^. The deviation in the orientation angle and aspect ratio of ECs decreased 6 h after the start of the experiment (Fig. 5b,c). The changes of orientation and coherency of actin filaments also indicated the EC alignment (Fig. S11). Morphological deformation is believed to be related to cell orientation in the flow direction^37,38^. Microscopic observation by immunofluorescence staining showed that the ratio of VE-cadherin per cell decreased under the hypoxic condition without flow exposure, while it increased after flow exposure (Fig. 5d). In our previous research^31^, we observed that hypoxic exposure to an oxygen concentration of < 3% caused internalization of VE-cadherin from the cell membrane of ECs, weakening intercellular adhesion and increasing collective cell migration. However, in the present study we observed no distinct difference in migration speed at different oxygen conditions (Fig. 4b), possibly due to a slightly higher oxygen concentration of about 4% O_2_ in the H3_stat_ and H3_flow_ conditions (Fig. 3b). In addition, it has been reported that when ECs orient in response to flow exposure, VE-cadherin is first reduced, then later enhanced^39^. The present study similarly showed cell orientation to be in the flow direction and enhancement of VE-cadherin after 6 h of flow exposure. Hence, under hypoxic exposure, intercellular adhesion by VE-cadherin is loosened and flow-induced cell orientation is actively promoted, then after cell orientation is complete, VE-cadherin is enhanced and collective cell migration is reduced. These results indicate that the collective migration and morphological changes of ECs could be dependent on the decrease of VE-cadherin expression by the hypoxic exposure. An ischemia–reperfusion event reportedly causes transient hypoxia and reoxygenation, as well as variations in fluid shear stress, and triggers the production of toxic substances such as reactive oxygen species, leading to disorders such as organ dysfunction and hemorrhage^40^. In order to establish prevention methods for these conditions, the developed microfluidic platform will be a useful tool for elucidating the cellular dynamics of ECs in response to hypoxic and flow exposure.

In the present study, we measured collective cell migration immediately after the microfluidic device was set in the stage incubator. However, about 1 h was required to stabilize the oxygen concentration and temperature at the target values. In addition, we limited the experiments to a maximum of 6 h, which is too short for the morphological changes of the cells to be complete^41^. Further investigation on the cellular dynamics happening both in minutes and days are required using a microfluidic platform with a fast time response for environmental control and a tolerance for long-duration experiments. The orientation of ECs reportedly takes more time than their elongation, and ECs become more elongated upon exposure to higher shear stress^42^. The observation of ICAM-1 expression indicated that EC monolayer became functionally different by hypoxic and/or flow exposure for 6 h (Fig. S12), but it requires further investigation with longer experiments. The dependence of morphological changes of ECs on the level and temporal variation of oxygen concentration and fluid shear stress also needs to be investigated. Moreover, the channel width of 500 µm in the present microfluidic device might have constrained collective cell migration^43,44^. Observation of cell migration free from the geometrical constraint of the channel would be instructive. By replicating the experimental settings used in former experiments^29,30^, only the ECs on the bottom glass coverslip of the media channel were investigated. Investigations of the changes of cell behaviors and endothelial permeability at the interface between gel and media channels will be examined in the future work. Elucidation of the intracellular signaling mechanism that leads to enhanced motility after sensing hypoxic or flow exposure is another future research topic.

## Conclusion

We developed a microfluidic platform that allows the observation of cells while simultaneously subjecting them to hypoxia and fluid flow by combining a microfluidic device and a flow channel for adjusting the oxygen concentration in the cell culture medium. The collective migration and morphological changes of ECs in a monolayer depended on the oxygen concentration, showing that simultaneous exposure to hypoxia and flow enhanced collective cell migration and promoted morphological changes, with the cells orienting toward the flow direction in the early phase of exposure. The developed microfluidic platform is useful for investigating the cellular dynamics of ECs in a vascular microenvironment.

## Methods

### Microfluidic platform for oxygen control

To reproduce an in vivo vascular microenvironment, we developed an experimental platform by integrating a microfluidic device and a flow channel that allows adjustment of the initial oxygen concentration in a cell culture medium. This microfluidic device controls the oxygen concentration under static conditions (without flow of cell culture medium) and was reported by us previously^28^ (Fig. 1). The device is 35 mm in diameter and ~ 6 mm thick, and was fabricated from poly(dimethylsiloxane) (PDMS), a polycarbonate (PC) film, and a glass coverslip. A gel channel (1300 µm wide) which mimics ECM when filled with a hydrogel is located in the center of the device, and is flanked by a Y-shaped media channel (500 µm wide) through which cell culture medium is supplied. Cells can be seeded in either or both of gel and media channels to observe their migration in the gel^26–28^ or on the channel surface^29,30^ or to evaluate endothelial permeability at the interface of the channels^31^. Gas channels (500 µm wide) are located on both sides of the channels to supply gas mixtures at set oxygen concentrations. The media and gas channels are partitioned by a 150 µm thick PDMS film. The cell culture medium and gas mixtures do not directly contact each other, and the oxygen concentration in the device is controlled and maintained by continuous gas exchange through the PDMS. Each channel is 150 µm high. The effects of oxygen infusion from the atmosphere into the device is minimized using a PC film (32 mm in diameter and 0.5 mm thick) with low gas permeability embedded in the device 0.5 mm from the bottom glass coverslip.

During fabrication of the device, the channel pattern was formed on a silicon wafer by photolithography, then transferred to PDMS by soft lithography^28^. PDMS (Sylgard 184 Silicone Elastomer Kit; Dow Corning, USA) was prepared by mixing the base and curing agent at a ratio of 10:1. After degassing, it was poured over a SU-8 channel pattern on the silicon wafer to a thickness of 0.5 mm and cured in an oven at 60 °C for > 4 h. A PC film with open holes at the locations of the inlets and outlets of each channel (3.2 mm in diameter for the media and gas channels and 1.6 mm in diameter for the gel channels) was placed on the cured PDMS layer. A fresh PDMS layer was then poured over the PDMS layer and the PC film to a thickness of 6 mm and cured in the oven overnight. The cured PDMS mold was then peeled off from the wafer and cut into a circle 35 mm in diameter. Inlets and outlets for each channel (2 mm in diameter for the media and gas channels and 1 mm in diameter for the gel channels) were punched into the PDMS mold to access the channels. The channel-patterned PDMS and a glass coverslip were plasma-treated with a plasma cleaner (PDC-32G; Harrick Plasma, USA) and bonded together to form microchannels. Immediately afterwards, 1 mg/ml poly-D-lysine (PDL) hydrobromide solution (P7886; Sigma-Aldrich, USA) was infused into the media and gel channels to aid adhesion of the gel and cells on the channel surfaces, and the device was placed in an incubator (5% CO_2_, 37 °C) for 4 h. After aspirating the PDL solution, the media and gel channels were washed with sterilized water twice and the device was dried in the oven overnight. Next, type I collagen solution (354236; Corning, USA), adjusted to 2.5 mg/ml at pH 7.4, was injected into the gel channel and polymerized in the incubator for 40 min. EGM-2 cell culture medium (CC-3162; Lonza, Switzerland) was then infused into the media channel to prevent the gel from drying. Moreover, to avoid disruption of the collagen gel in the gel channel during the flow exposure experiments, the inlet and outlet of the gel channel were covered by bonding a 9 × 9 mm glass coverslip. The device was placed in the incubator overnight to mechanically stabilize the collagen gel. Just before seeding the cells in the media channel, the surface of the channel was coated with 2.0 mg/ml Matrigel solution (354234; Corning) to enhance cell adhesion.

Controlling the oxygen concentration in the device became difficult when the cell culture medium flowed in the media channel due to inadequate oxygen exchange between the channels. Hence, the oxygen concentration in the cell culture medium was initially adjusted to an intended value, while the cell culture medium travelled through the flow channel placed in a stage incubator (INUBSF-ZILCS; Tokai HIT, Japan) (5% CO_2_, 37 °C) before being supplied to the media channel. The flow channel was 1 mm wide, 1 mm high, and 500 mm long. The channel was designed to operate while placed in a water bath area of the stage incubator (Fig. 1 and Supplementary Fig. S1). This allowed temperature control of the cell culture medium at 37 °C, as well as oxygen concentration control. The flow channel was fabricated by creating a mold with the channel pattern by cutting acrylonitrile–butadiene–styrene (ABS) resin with a 3D modelling machine (monoFab SRM-20; Roland DG, Japan). PDMS was poured over the mold to a height of 3 mm, cured in the oven for > 4 h, then peeled off from the mold. After 2 mm diameter holes were punched at the inlets and outlets of the flow channel, the PDMS was bonded to a 0.5 mm-thick PDMS sheet with the same shape using a thin layer of fresh PDMS, and placed in the oven overnight.

The cell culture medium was circulated using a tubing pump (FP-100-2; AS ONE, Japan). The flow channel, media channel in the device, and a reservoir were connected with tubing (Fig. 1). Gas mixtures supplied to the gas channels in the device and the stage incubator were prepared by mixing oxygen, carbon dioxide, and nitrogen with gas blenders (3MFC GAS MIXER; KOFLOC, Japan, and MU-3405; HORIBA STEC, Japan), maintaining 5% CO_2_. The gas mixtures were humidified by bubbling through water before being supplied to the gas channels. The experimental conditions were named according to the oxygen concentration in the gas mixture, and the flow of the cell culture medium (Table 1). The normoxic condition at 21% O_2_ was termed “N”. A hypoxic condition generated by supplying a gas mixture was termed “H” accompanied with the value of the oxygen concentration* c*_g_ in the gas mixture supplied to the gas channels. The conditions with and without flow exposure are noted with the subscripts “flow” and “stat”, respectively. As for the experiments under hypoxic and flow exposure conditions, the oxygen concentrations *c*_i_ and *c*_m_ in the stage incubator and the cell culture medium, respectively, were adjusted at the same value based on computational results. Moreover, the hypoxic static conditions generated by simultaneously adjusting the oxygen concentrations *c*_g_, *c*_i_, and *c*_m_ for the reference in validation experiment were termed “R”. The directions orthogonal and parallel to the channels in the device were defined as the *x* and *y*-directions, respectively, and the vertical direction was defined as the *z*-direction (Fig. 1). Consequently, the flow direction in the media channels was in the -*y*-direction.

**Table 1:** Experimental conditions and settings: oxygen concentration, *c*_g_ and *c*_i_, in the supplied gas mixtures to the gas channels and the stage incubator, respectively, and time period in validation experiment or cellular experiment.

Condition	O_2_ concentration [%]		Time period [h]	
	Gas channels *c*_g_	Stage incubator *c*_i_	Validation expt	Cellular expt
N_stat_	21	21	2	6
H10_stat_	10	21	–	–
H5_stat_	5	21	–	–
H3_stat_	3	21	5	6
H1_stat_	1	21	–	–
H0_stat_	0	21	5	–
N_flow_	21	21	–	6
H3_flow_	3	3	3	6
H0_flow_	0	0	8	–
R0–R10	0–10	0–10	≥ 2	–

The normoxic condition is termed “N”, and a hypoxic condition is termed “H” accompanied with the value of *c*_g_. The conditions with and without flow exposure are noted with the subscripts “flow” and “stat”, respectively. The hypoxic static conditions for the reference in validation experiment are termed “R”.

### Computational analysis for oxygen concentration control

The flow field in the media and gas channels and the oxygen condition generated in the device were analyzed by 3D numerical simulation using commercial multiphysics software (COMSOL Multiphysics ver. 5.5, COMSOL AB, Sweden)^27^. In the flow analysis, the Navier–Stokes equations and the equation of continuity were solved as the governing equations. The physical properties of the cell culture medium, gas mixture, hydrogel, PDMS, and PC film are summarized in Table 2. The flowrates or the average flow speed of the cell culture medium and gas mixture were applied with reference to the Péclet number *Pe*_m_ and *Pe*_g_, respectively. Here, *Pe* is a nondimensional value representing the ratio between convection and diffusion:

$$Pe = \frac{UL}{D} ,$$

where *U* is the reference velocity, *L* is the reference length (the channel width), and *D* is the diffusion coefficient of oxygen in each material. A zero pressure condition was applied on all outlets of the media and gas channels. A no-slip condition was applied on the channel surface. The convection–diffusion equation was solved in the following analysis for the distribution of the oxygen concentration. Based on the oxygen concentration* c*_i_ in the stage incubator or the atmosphere surrounding the device and the solubility of oxygen *S* (Table 2), the initial distributions *C* of the oxygen in PDMS, PC, and the cell culture medium were set in the unit of mM based on Henry’s law. At the interfaces between PDMS and the cell culture medium or gel, a partition condition was applied, which balanced the mass flux of oxygen to satisfy the continuity of the partial pressure of oxygen:

$$\frac{{C}_{\mathrm{PDMS}}}{{S}_{\mathrm{PDMS}}} = \frac{{C}_{\mathrm{channel}}}{{S}_{\mathrm{channel}}} ,$$

where *C*_PDMS_ and *S*_PDMS_ are the oxygen distribution and solubility in PDMS, respectively, and *C*_channel_ and *S*_channel_ are those in the media and gel channels. A no-flux condition was applied to the bottom surface, which was covered by a glass coverslip. Gas mixtures with an oxygen concentration *c*_g_ ranging from 0 to 21% were assumed to be supplied to both gas channels (Table 1). Regarding to the hypoxic and flow exposure conditions, four different methods A-D were computationally examined by dealing with the condition H0_flow_ (Table 3). The oxygen concentrations *c*_i_ and *c*_m_ in the stage incubator and the cell culture medium, respectively, were independently changed in addition to gas supply at the oxygen concentration *c*_g_ to the gas channels. The computed values *C* of oxygen concentration were finally converted to percentages *c* by referring to the solubility of oxygen in each material for easy understanding. The computational grid for the numerical simulation consisted of 1,603,638 elements, as determined by a grid independence test for the computed oxygen concentration and fluid shear stress τ.

**Table 2:** Physical properties of each component and parameters.

	Medium	Gas	Gel	PDMS	PC film
Density, ρ [kg/m^3^]	1.0 × 10^3^	1	1.0 × 10^3^		
Viscosity, µ [Pa s]	1.0 × 10^−3^	1.0 × 10^−5^			
Diffusivity of oxygen, *D* [m^2^/s]	2.0 × 10^−9^	2.0 × 10^−5^	2.0 × 10^−9^	4.0 × 10^−9^	2.0 × 10^−12^
Solubility of oxygen at 21% O_2_, *S* [mM]	0.218		0.218	1.25	1.25
Péclet number, *Pe*	≤ 10,000	≤ 1000			
Average velocity, *U* [m/s]	≤ 4.0 × 10^−2^	≤ 40			
Flow volume, *Q* [ml/min]	≤ 1.8 × 10^−1^	≤ 1.8 × 10^2^

**Table 3:** Computational settings in consideration to generate the hypoxic condition H0_flow_: oxygen concentrations *c*_g_ and *c*_i_ in the supplied gas mixtures to the gas channels and the stage incubator, respectively, and oxygen concentration *c*_m_ in the cell culture medium.

Method	O_2_ concentration [%]		
	Gas channels *c*_g_	Stage incubator *c*_i_	Medium *c*_m_
A	0	21	21
B	0	0	21
C	0	21	0
D	0	0	0

### Validation experiment of oxygen concentration control

Control of the oxygen concentration with the microfluidic platform developed with the aid of 3D numerical simulation was validated. The oxygen concentration was determined by measuring the phosphorescence intensity of an oxygen-sensing film, which is quenched by oxygen, and converting the intensity into the oxygen concentration^45^. Platinum(II) 5,10,15,20-tetrakis-(2,3,4,5,6-pentafluorophenyl)porphyrin (PtTFPP, Por-Lab, Porphyrin-Laboratories, Germany) of 17 mg was first dissolved in 5 ml chloroform and mixed with the 3.5 g PDMS (a mixture of the base and curing agent at a ratio of 4:1). The oxygen-sensing film was then formed by spin coating the PDMS containing PtTFPP on a glass coverslip at 500 rpm for 2 min. The coated glass coverslip was bonded to the bottom of the microfluidic device after plasma treatment in place of the glass coverslip. The other processes for preparing the microfluidic platform were the same as those described above. The microfluidic device and the flow channel were placed in the stage incubator mounted on a fluorescence microscope (EVOS FL Cell Imaging System; Life Technologies, USA). The gas mixtures at predefined oxygen concentrations were supplied to the gas channels and the stage incubator, and the cell culture medium was circulated by the tubing pump (Fig. 1) to generate the conditions summarized in Table 1. After the phosphorescence from the oxygen-sensing film reached a steady state under each condition, 1280 × 960 pixel fluorescence microscopic images were obtained. A 1300 × 100 µm region of interest (ROI) was set in each microscopic image. The average phosphorescence intensity was calculated in each area by dividing the ROI every 100 µm in the *x*-direction and converting to oxygen concentration using the Stern–Volmer equation^45^:

$$c\left(x\right)=\frac{\left(\frac{{{\overline{I} }^{^{\prime}}}_{\mathrm{A}}\left(x\right)-{{\overline{I} }^{^{\prime}}}_{\mathrm{BG}}\left(x\right)}{{\overline{I} }^{^{\prime}}\left(x\right)-{{\overline{I} }^{^{\prime}}}_{\mathrm{BG}}\left(x\right)}-1\right)}{{K}_{q}\left(x\right)}\times 100 ,$$

where *Ī’*(*x*) and *Ī’*_A_(*x*) are phosphorescence intensities under arbitrary and anoxic conditions, respectively, *Ī’*_BG_(*x*) is the background noise due to autofluorescence of the cell culture medium, and *K*_*q*_ is the quenching constant. The value of *K*_*q*_ was derived by using the phosphorescence intensities under the conditions R0-R10 and N_stat_, in which the same gas mixture was supplied to the gas channels and the stage incubator, respectively. The oxygen concentration inside the device was measured using three of the described microfluidic devices.

### Cellular experiment

Collective migration and morphological changes of ECs formed a confluent monolayer on the bottom of the media channel were measured by controlling the oxygen concentration and media flow^29,30^. Human umbilical vein endothelial cells (HUVECs) (C2517A; Lonza, Switzerland) below the 9th passage were used in the experiment. ECs cultured on cell culture dishes just before confluency were harvested by trypsinization. The cell suspension was adjusted to a cell density of 5 × 10^6^ cells/ml and introduced into the media channels. An EC monolayer was formed by culturing the cells until 100% confluency and changing the cell culture medium daily^30,31^. The device was then placed in the stage incubator mounted on the fluorescence microscope, and the EC monolayer was exposed to four different conditions combining oxygen and flow conditions (i.e., N_stat_, H3_stat_, N_flow_, and H3_flow_ in Table 1). These conditions were chosen by considering ordinary normoxic experimental conditions of 21% O_2_ and physiologically relevant hypoxic conditions of < 5% O_2_^18^. In the conditions with flow exposure, fluid shear stress was applied to the ECs by circulating the cell culture medium in the media channel at *Q*_m_ = 100 µl/min. Here, the actual viscosity and density of the cell culture medium at 37 °C were measured by using a vibration-type viscometer (VM-10A; SEKONIC, Japan) and an electronic balance (ASP413; AS ONE, Japan); the measured values were 0.757 × 10^−3^ Pa s and 0.979 × 10^3^ kg/m^3^, respectively. Hence, the cell culture medium generated a fluid shear stress τ of 0.72 Pa at the center of the bottom of the media channel as shown later with computational results, which was in a physiological range^1^. Phase-contrast microscopic images of the EC monolayer were obtained every 10 min for 6 h after the start of each experiment. The migration speed of the cells was measured by PIV with time-lapse microscopic phase-contrast images obtained every 30 min using JPIV open-source software^46^. A 440 × 825 µm (512 × 960 pixel) rectangular region in the media channel was divided into small ROIs of 6.88 × 6.88 µm (8 × 8 pixel). The cross-correlation function of each ROI between two sequential time points provided the displacement and was converted to the migration velocity of the cells. The migration velocity was calibrated by eliminating device drifting, estimated by displacement of the PDMS pillars supporting the gel channel.

After the 6-h cellular experiment under each condition, the cells were fixed with 4% paraformaldehyde phosphate buffer solution (163–20145; Wako Pure Chemical Industries, Japan) for 10 min and permeabilized with 0.1% Triton X-100 (T9284; Sigma-Aldrich) in phosphate-buffered saline (PBS) (P5119; Sigma-Aldrich) for 5 min. The cells were then blocked with 1% Block Ace (BA, DS Pharma Biomedical, Japan) in Dulbecco’s PBS (DPBS, D8537; Sigma-Aldrich) (BA-DPBS) for 30 min to prevent nonspecific absorption of the antibodies described below. VE-cadherin or ICAM-1 was labeled with mouse monoclonal antibody (sc-9989; Santa Cruz Biotechnology, USA, or 62133; Cell Signaling Technology, USA) at a dilution of 1:200 or 1:500 in BA-DPBS for 1 h, followed by staining with Alexa Fluor 488 goat-anti-mouse secondary antibody (A11001; Thermo Fisher Scientific, USA) at a dilution of 1:200 or 1:100 in PBS for 1 h, respectively. Cell nuclei and actin filaments were stained with DAPI (D21490; Thermo Fisher Scientific) at 1 μg/ml and Alexa Fluor 594 phalloidin (A12381; Thermo Fisher Scientific), respectively. Immunofluorescence staining was conducted at room temperature. The cells were washed twice with DPBS between each step. Twenty microscopic images of the cells on the bottom of the media channel on the horizontal plane (*xy*-plane) were taken at 0.6 µm intervals in the vertical (*z*-axial) direction using a confocal laser scanning microscope (LSM800; Carl Zeiss Microscopy, Germany), and the maximum intensity of the images was projected onto the *xy*-plane. The projected images were then analyzed using open-source software (ImageJ, National Institutes of Health, USA) to extract the outer and inner outlines of VE-cadherin surrounding each cell. The cell shape was approximated to be ellipsoidal based on the outer outline of VE-cadherin. Then, the orientation angle formed by the major axis and the flow direction (the -*y*-direction), and the aspect ratio between the lengths of the major and minor axes, were quantified. Furthermore, to evaluate cell–cell junctions, the area *A*_cad_ of VE-cadherin of each cell was obtained as the difference of the areas *A*_out_ and *A*_in_ surrounded by the extracted outer and the inner outlines, and the ratio of the VE-cadherin area to the total cell area, A^*^_cad_ (= *A*_cad_/*A*_out_), was calculated^47^. The fiber orientation and coherency in the cells were measured by analyzing the images of actin filaments with ImageJ with its built-in plugin, OrientationJ^48^. The expression of ICAM-1 was evaluated by dividing the area of ICAM-1 (pixels of intensity value 30–255) by the area of ECs^49^.

The migration velocities of the cells were measured with four of the described microfluidic devices for each condition. Microscopic observation of the cells with immunofluorescence staining was performed with three microfluidic devices for each condition, and five arbitrary locations were observed in each device. Cell morphology and VE-cadherin were evaluated by choosing six and four cells randomly in each microscopic image, respectively, resulting in 90 and 72 cells in total. Actin filaments and ICAM-1 were evaluated with the 15 images in total. The orientation angles of the cells were compared between conditions by the Kolmogorov–Smirnov test. Significant differences in the aspect ratio, the ratio A^*^_cad_ of the VE-cadherin area to the total area of the cells, the orientation and coherency of actin filaments, and the ICAM-1expression area ratio were assessed by two-way analysis of variance (ANOVA) followed by post hoc Tukey’s test. In each test, statistical significance was inferred at *P* < 0.05.

## Supplementary Information

Supplementary Information 1.

Supplementary Video 1.

Supplementary Video 2.

Supplementary Video 3.

Supplementary Video 4.

## Data Availability

The data presented in this study are available from the corresponding author upon reasonable request.

## References

[1] Gray KM, Stroka KM (2017). Vascular endothelial cell mechanosensing: New insights gained from biomimetic microfluidic models. Semin. Cell Dev. Biol..

[2] Ohta M, Sakamoto N, Funamoto K, Wang Z, Kojima Y, Anzai H (2022). A review of functional analysis of endothelial cells in flow chambers. J. Funct. Biomater..

[3] Ohashi T, Sato M (2005). Remodeling of vascular endothelial cells exposed to fluid shear stress: Experimental and numerical approach. Fluid Dyn. Res..

[4] Sato M, Levesque MJ, Nerem RM (1987). Micropipette aspiration of cultured bovine aortic endothelial cells exposed to shear stress. Arteriosclerosis.

[5] Ilina O, Friedl P (2009). Mechanisms of collective cell migration at a glance. J. Cell Sci..

[6] Trepat X, Chen ZZ, Jacobson K (2012). Cell migration. Compr. Physiol..

[7] Haeger A, Wolf K, Zegers MM, Friedl P (2015). Collective cell migration: Guidance principles and hierarchies. Trends Cell Biol..

[8] Hakim V, Silberzan P (2017). Collective cell migration: A physics perspective. Rep. Prog. Phys..

[9] Szabo A (2010). Collective cell motion in endothelial monolayers. Phys. Biol..

[10] Scarpa E, Mayor R (2016). Collective cell migration in development. J. Cell Biol..

[11] Montell DJ (2008). Morphogenetic cell movements: Diversity from modular mechanical properties. Science.

[12] Friedl P, Gilmour D (2009). Collective cell migration in morphogenesis, regeneration and cancer. Nat. Rev. Mol. Cell Biol..

[13] Lamalice L, Le Boeuf F, Huot J (2007). Endothelial cell migration during angiogenesis. Circ. Res..

[14] Bentley K (2014). The role of differential VE-cadherin dynamics in cell rearrangement during angiogenesis. Nat. Cell Biol..

[15] Hayer A (2016). Engulfed cadherin fingers are polarized junctional structures between collectively migrating endothelial cells. Nat. Cell Biol..

[16] Michaelis UR (2014). Mechanisms of endothelial cell migration. Cell Mol. Life Sci..

[17] Keeley TP, Mann GEE (2019). Defining physiological normoxia for improved translation of cell physiology to animal models and humans. Physiol. Rev..

[18] Carreau A, El Hafny-Rahbi B, Matejuk A, Grillon C, Kieda C (2011). Why is the partial oxygen pressure of human tissues a crucial parameter? Small molecules and hypoxia. J. Cell Mol. Med..

[19] Muz B, de la Puente P, Azab F, Azab AK (2015). The role of hypoxia in cancer progression, angiogenesis, metastasis, and resistance to therapy. Hypoxia (Auckl).

[20] Eltzschig HK, Carmeliet P (2011). Hypoxia and inflammation. N. Engl. J. Med..

[21] Krock BL, Skuli N, Simon MC (2011). Hypoxia-induced angiogenesis: Good and evil. Genes Cancer.

[22] Halldorsson S, Lucumi E, Gomez-Sjoberg R, Fleming RMT (2015). Advantages and challenges of microfluidic cell culture in polydimethylsiloxane devices. Biosens. Bioelectron..

[23] Wan L, Neumann CA, LeDuc PR (2020). Tumor-on-a-chip for integrating a 3D tumor microenvironment: Chemical and mechanical factors. Lab Chip.

[24] Byrne MB, Leslie MT, Gaskins HR, Kenis PJA (2014). Methods to study the tumor microenvironment under controlled oxygen conditions. Trends Biotechnol..

[25] Wu HM (2018). Review of microfluidic cell culture devices for the control of gaseous microenvironments in vitro. J. Micromech. Microeng..

[26] Funamoto K (2012). A novel microfluidic platform for high-resolution imaging of a three-dimensional cell culture under a controlled hypoxic environment. Lab Chip.

[27] Koens R (2020). Microfluidic platform for three-dimensional cell culture under spatiotemporal heterogeneity of oxygen tension. APL Bioeng..

[28] Yoshino D, Funamoto K (2019). Oxygen-dependent contraction and degradation of the extracellular matrix mediated by interaction between tumor and endothelial cells. AIP Adv..

[29] Hirose S (2021). P21-activated kinase regulates oxygen-dependent migration of vascular endothelial cells in monolayers. Cell Adh. Migr..

[30] Tabata Y (2019). Migration of vascular endothelial cells in monolayers under hypoxic exposure. Integr. Biol..

[31] Funamoto K (2017). Endothelial monolayer permeability under controlled oxygen tension. Integr. Biol..

[32] Abaci HE, Shen YI, Tan S, Gerecht S (2014). Recapitulating physiological and pathological shear stress and oxygen to model vasculature in health and disease. Sci. Rep..

[33] Angelini TE, Hannezo E, Trepat X, Fredberg JJ, Weitz DA (2010). Cell migration driven by cooperative substrate deformation patterns. Phys. Rev. Lett..

[34] Tsuboi H (1995). Flow stimulates ICAM-1 expression time and shear stress dependently in cultured human endothelial cells. Biochem. Biophys. Res. Commun..

[35] Ostrowski MA (2014). Microvascular endothelial cells migrate upstream and align against the shear stress field created by impinging flow. Biophys. J..

[36] Tkachenko E (2013). The nucleus of endothelial cell as a sensor of blood flow direction. Biol. Open.

[37] Kataoka N, Ujita S, Sato M (1998). Effect of flow direction on the morphological responses of cultured bovine aortic endothelial cells. Med. Biol. Eng. Comput..

[38] Tzima E (2005). A mechanosensory complex that mediates the endothelial cell response to fluid shear stress. Nature.

[39] Noria S, Cowan DB, Gotlieb AI, Langille BL (1999). Transient and steady-state effects of shear stress on endothelial cell adherens junctions. Circ. Res..

[40] Funamoto K, Ito T, Funamoto K, Velayo CL, Kimura Y (2017). Ultrasound imaging of mouse fetal intracranial hemorrhage due to ischemia/reperfusion. Front. Physiol..

[41] DeStefano JG (2017). Real-time quantification of endothelial response to shear stress and vascular modulators. Integr. Biol..

[42] Levesque MJ, Nerem RM (1985). The elongation and orientation of cultured endothelial cells in response to shear stress. J. Biomech. Eng..

[43] Vedula SR (2012). Emerging modes of collective cell migration induced by geometrical constraints. Proc. Natl. Acad. Sci. U. S. A..

[44] Doxzen K (2013). Guidance of collective cell migration by substrate geometry. Integr. Biol..

[45] Cochet-Escartin O (2021). Hypoxia triggers collective aerotactic migration in *Dictyostelium discoideum*. Elife.

[46] Vennemann, P. *JPIV*, https://eguvep.github.io/jpiv/. Accessed 26 September 2022.

[47] Seebach J (2007). Regulation of endothelial barrier function during flow-induced conversion to an arterial phenotype. Cardiovasc. Res..

[48] Rezakhaniha R (2012). Experimental investigation of collagen waviness and orientation in the arterial adventitia. Biomech. Model. Mechanobiol..

[49] Nam U (2020). Lipopolysaccharide-induced vascular inflammation model on microfluidic chip. Micromachines.

