# Supplementary material for: Microfluidic platform for the reproduction of hypoxic vascular microenvironments
Source: Sci Rep. 2023 Apr 3;13:5428. doi: 10.1038/s41598-023-32334-9 (PMC10070331; doi:10.1038/s41598-023-32334-9)
Supplement: Supplementary file 1 — Supplementary Information 1. [file 41598_2023_32334_MOESM1_ESM.pdf]

SUPPLEMENTARY MATERIAL

"Microfluidic platform for the reproduction of hypoxic vascular microenvironments" by Takahashi et al.

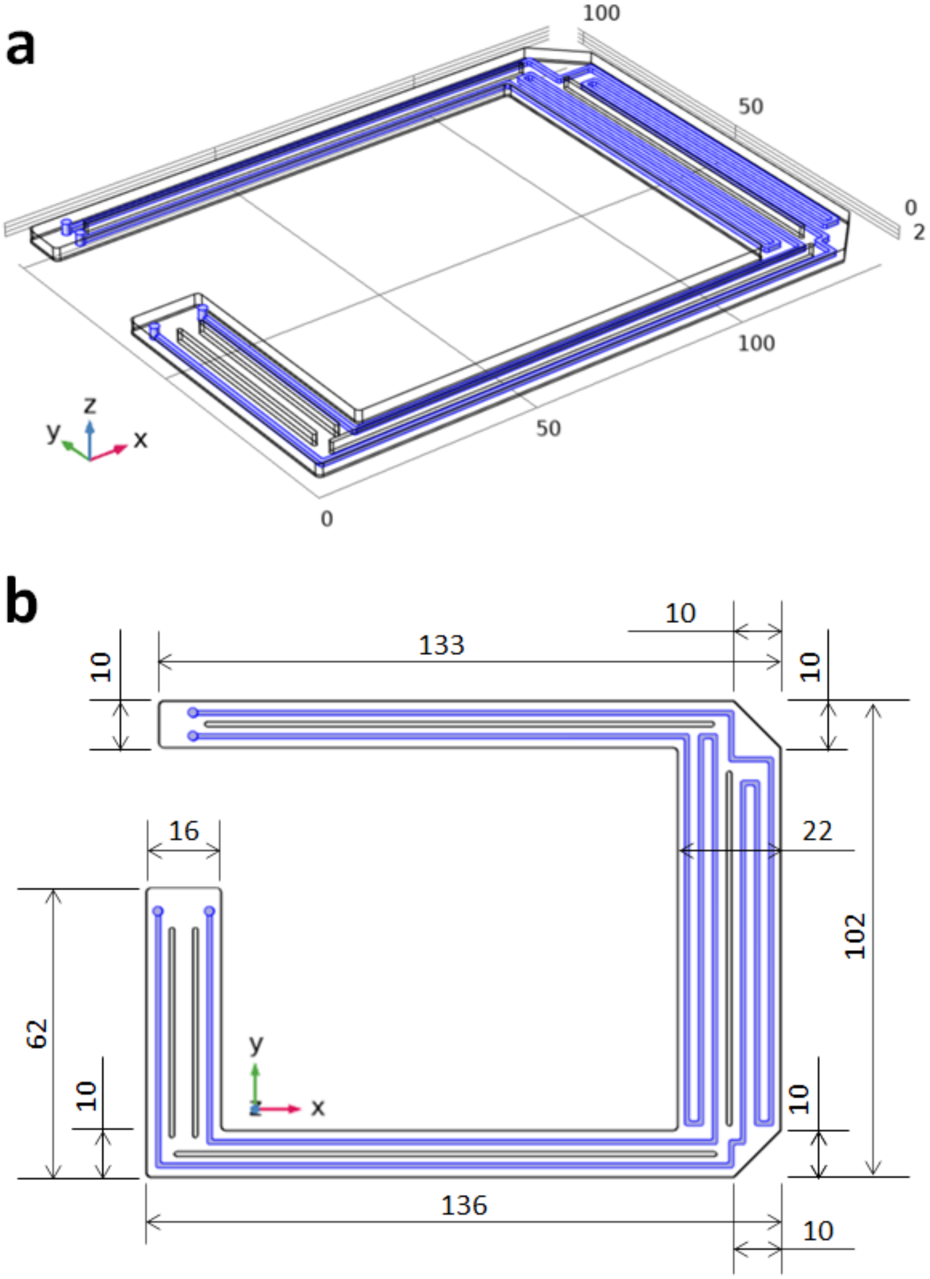

Fig. S1 Design of a flow channel to control of oxygen concentration in a cell culture medium: (a) the 3D view, and (b) the *xy*-plane view. Unit is mm. The flow channel was placed in a water bath area inside a stage incubator. The device is 3.5 mm height, and the two channels colored with blue are 1 mm wide, 1 mm high, and 500 mm long. Slits between the two channels are 1 mm wide.

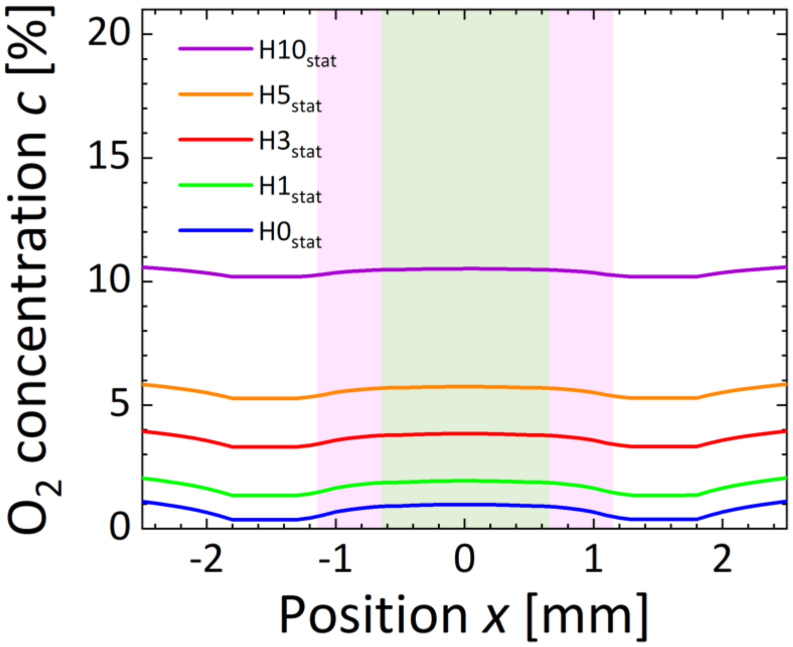

Fig. S2 Computational results of profiles of steady oxygen concentration c across the media and gel channels (y = 0 mm), generated by supplying a gas mixture containing 0, 1, 3, 5, or 10% O_2 to both gas channels without media flow ($H0_{stat}$, $H1_{stat}$, $H3_{stat}$, $H5_{stat}$, or $H10_{stat}$). The origin was set at the center of the gel channel. The x-direction was defined as the horizontal direction normal to the gel channel. Regions shaded with pink and green indicate sections of the media and gel channels, respectively.

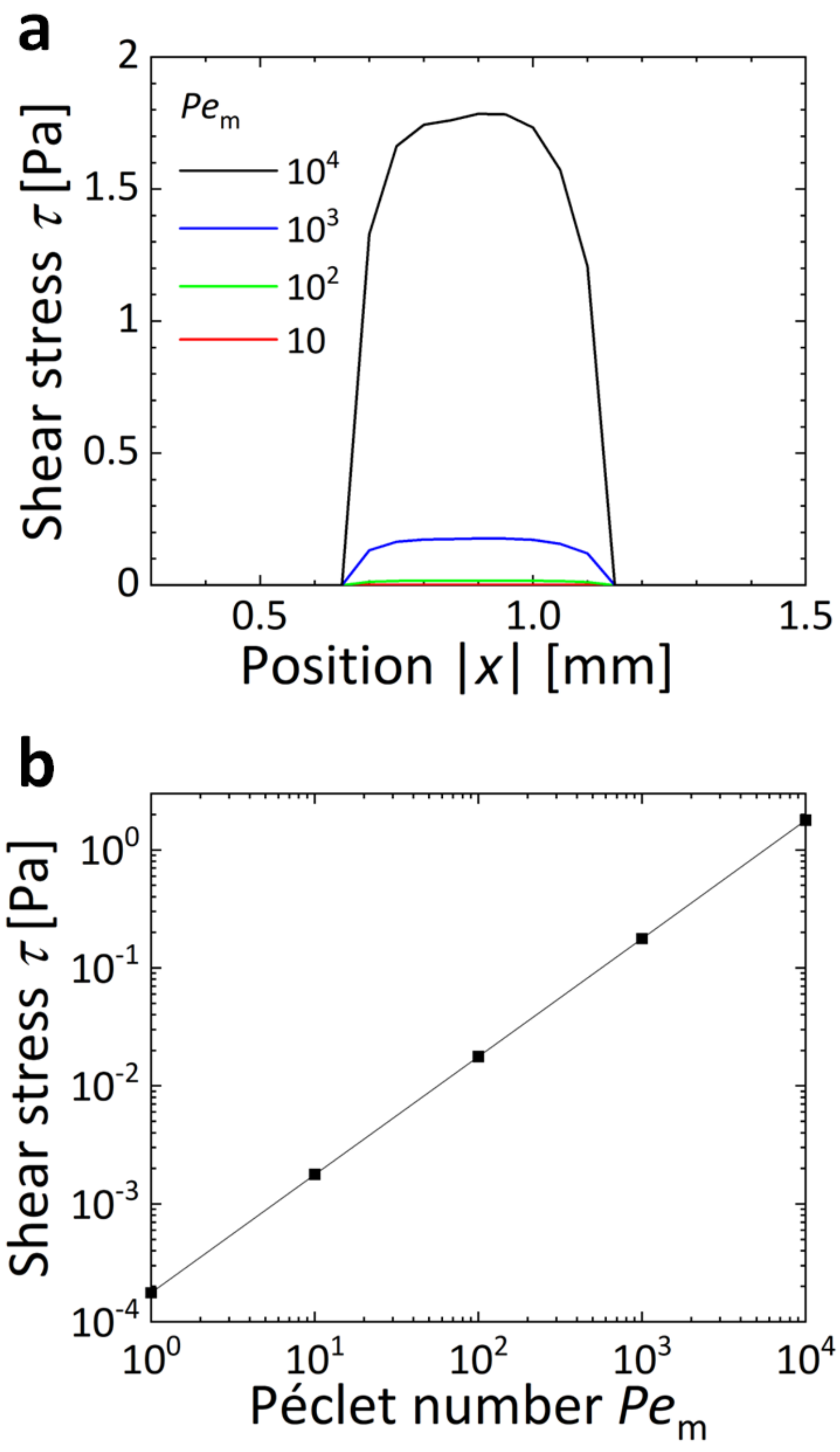

Fig. S3 Variation of fluid shear stress τ acting on the bottom surface of the media channel with Péclet numbers Pe_m for the medium flow: (a) the profile across the media channel (y = 0 mm, z = 0 mm), and (b) the value at the center of the media channel ($|x|$ = 0.9 mm, y = 0 mm, z = 0 mm). The viscosity and density of the cell culture medium were assumed as 1.0×10^{-3} Pa·s and 1.0×10^{3} kg/m^3, respectively, in the numerical simulation.

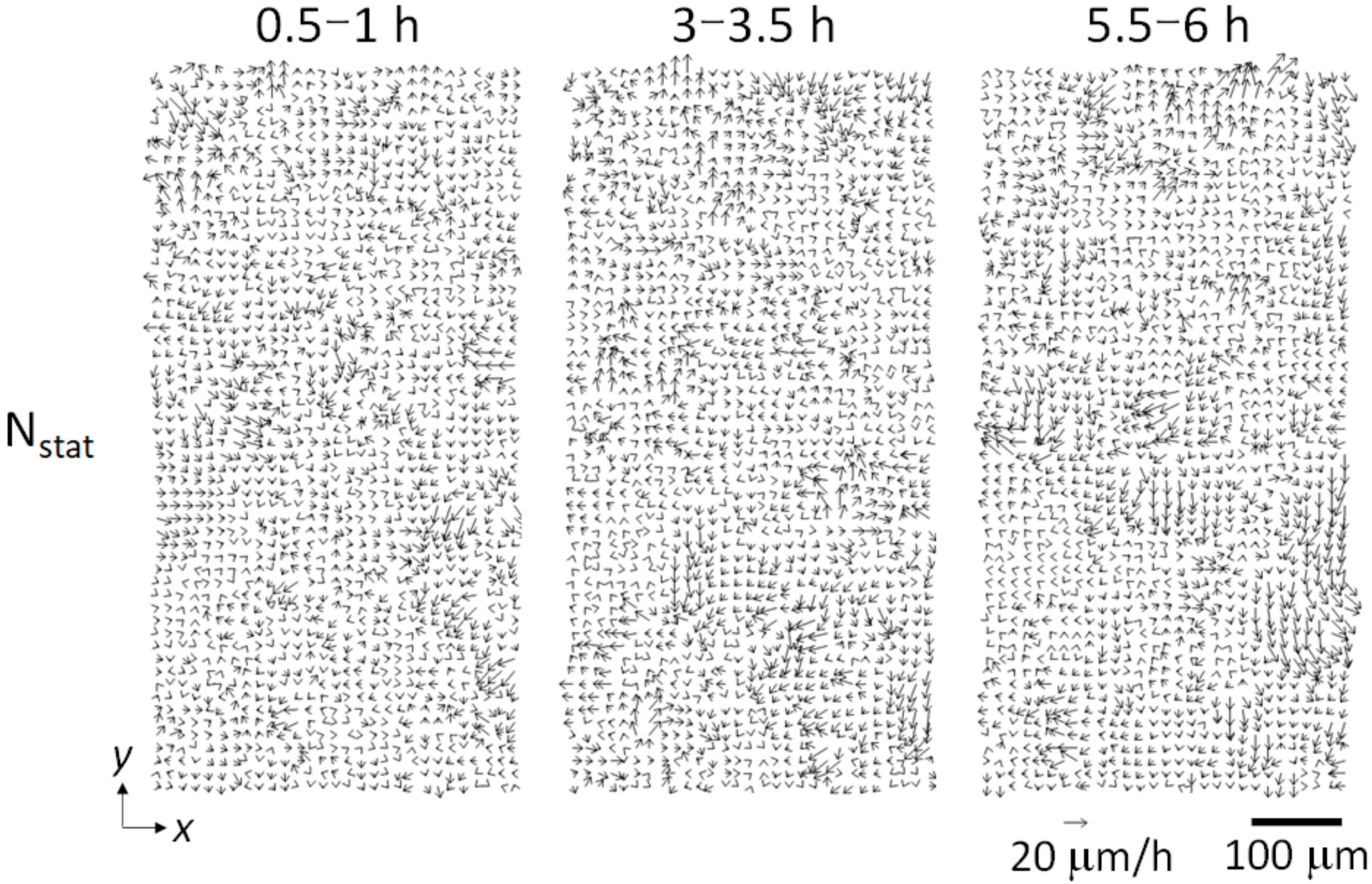

Fig. S4 Velocity vectors of collective migration of ECs in the monolayer under the normoxic condition N_{stat} without flow exposure.

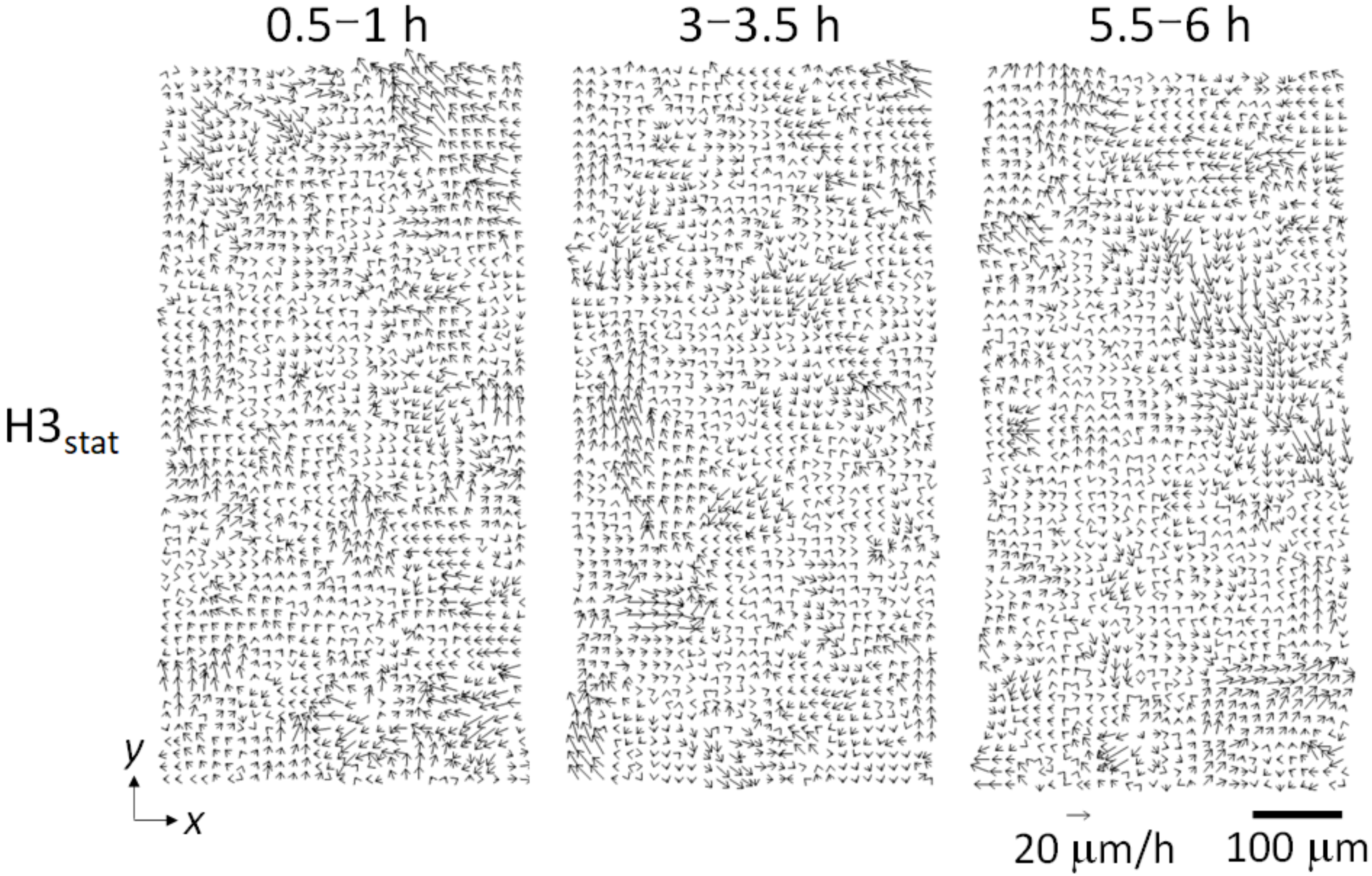

Fig. S5 Velocity vectors of collective migration of ECs in the monolayer under the hypoxic condition H3$_{stat}$ without flow exposure.

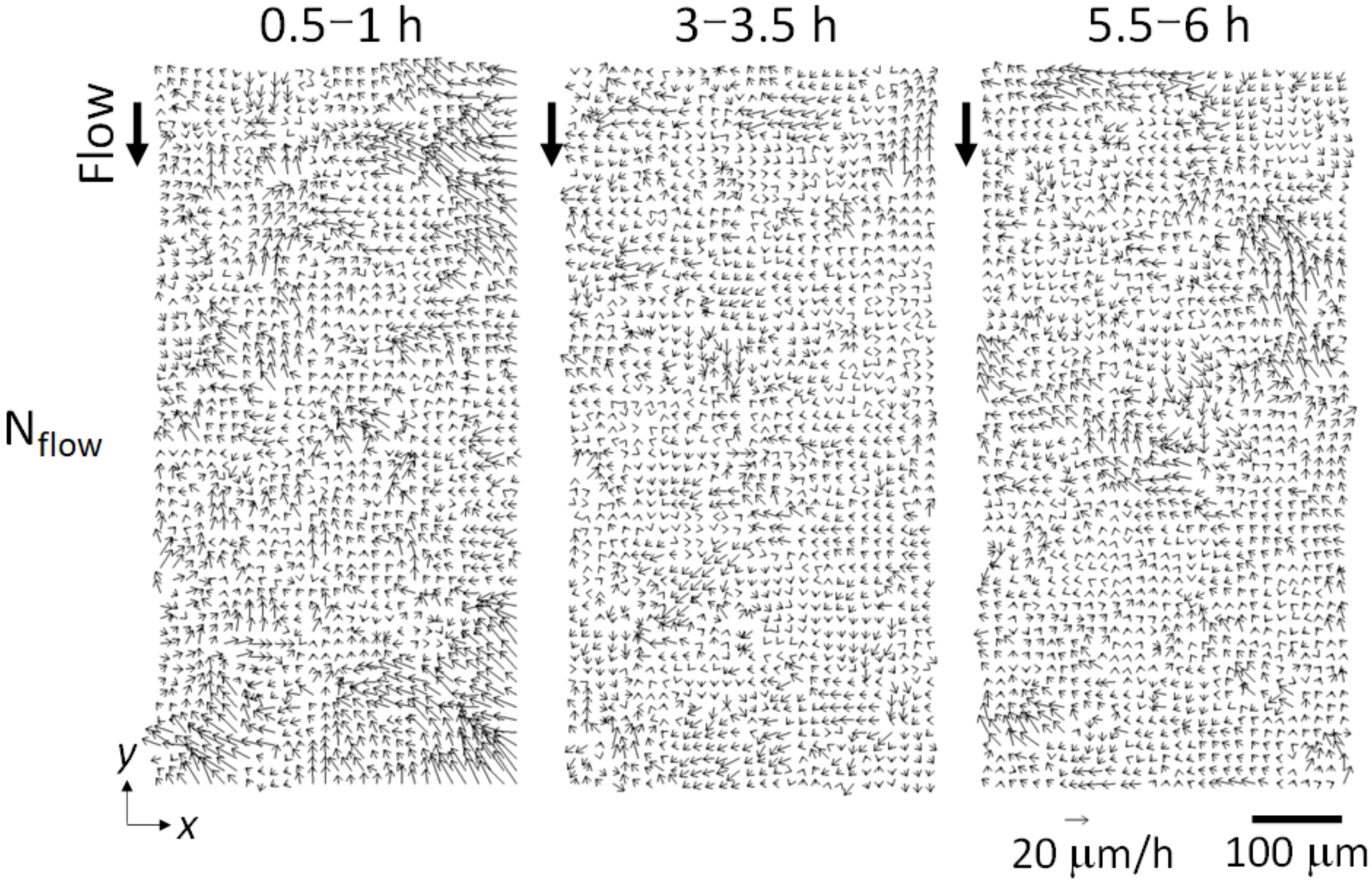

Fig. S6 Velocity vectors of collective migration of ECs in the monolayer under the normoxic condition N_{flow} with flow exposure in the -*y*-direction.

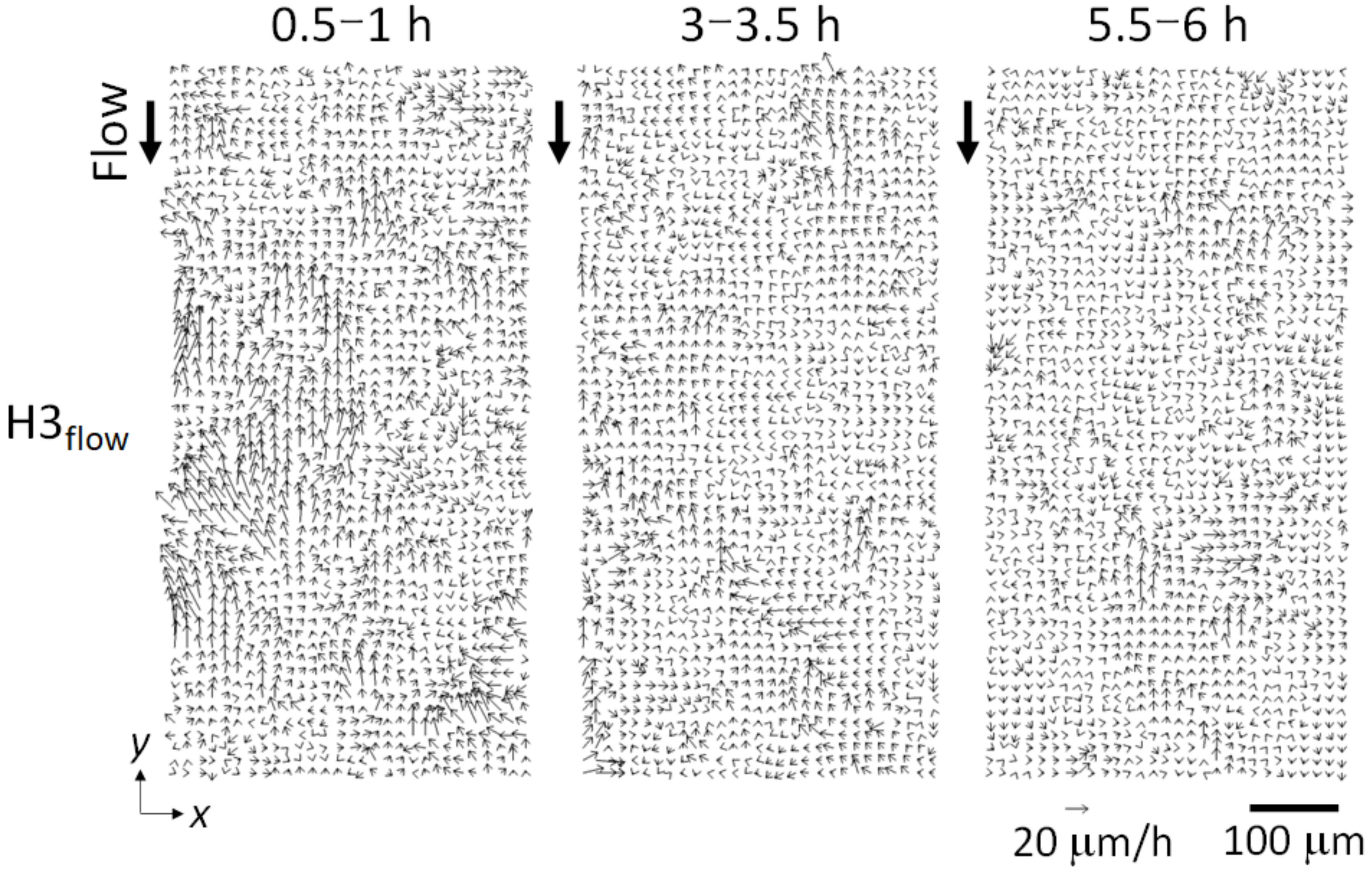

Fig. S7 Velocity vectors of collective migration of ECs in the monolayer under the hypoxic condition H3$_{flow}$ with flow exposure in the -*y*-direction.

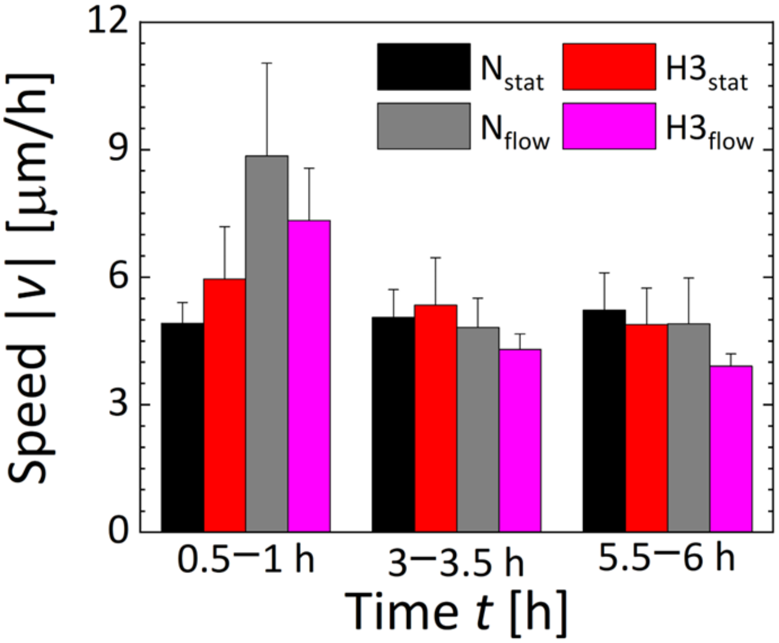

Fig. S8 Spatially averaged migration speed |*v*| of ECs between 0.5-1 h, 3-3.5 h and 5.5-6 h after exposing the cells to the four different oxygen and flow conditions N_{stat}, $H3_{stat}$, N_{flow}, and $H3_{flow}$. Error bars show the standard deviation.

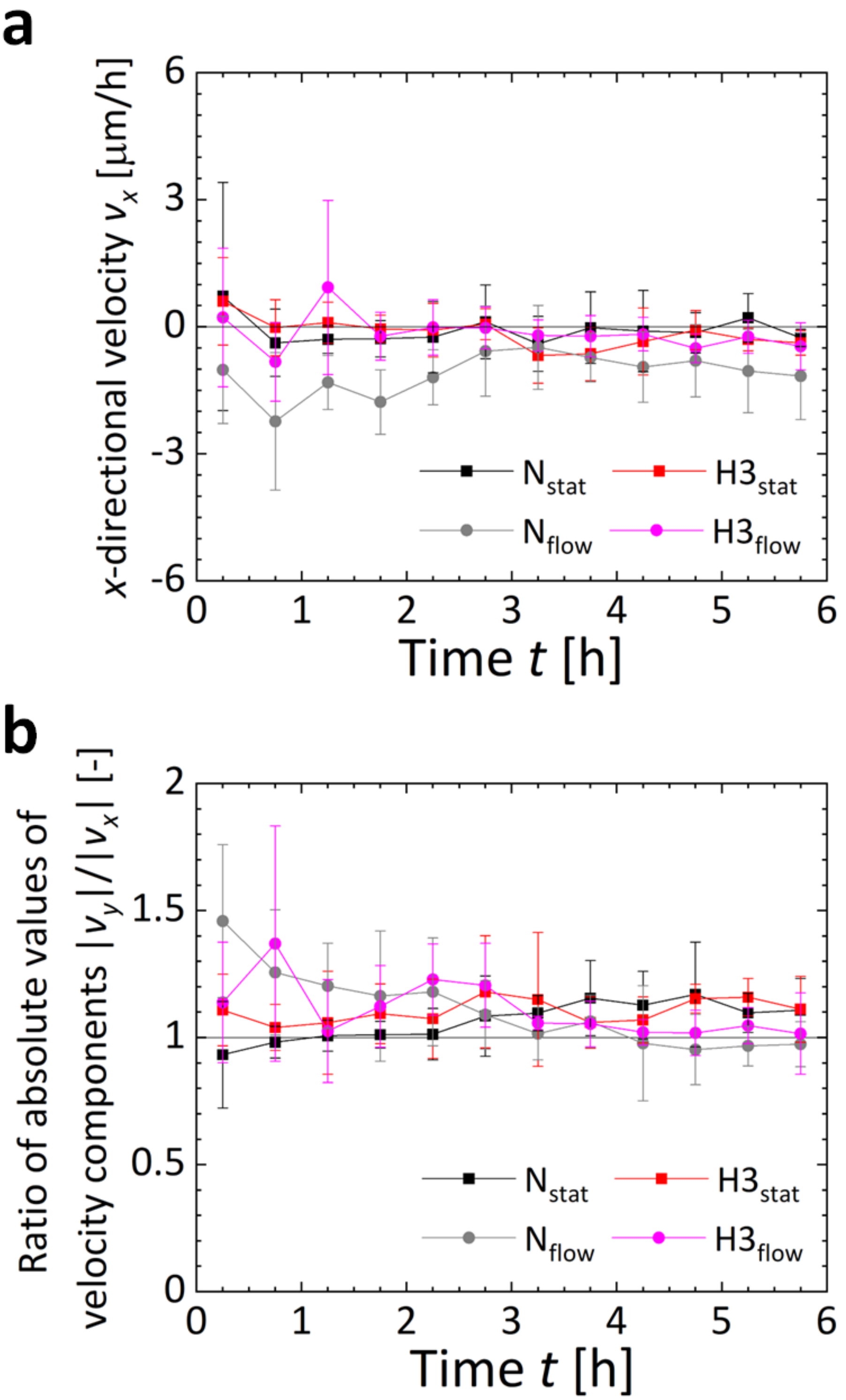

Fig. S9 Time variations of the spatially averaged x-directional velocity v_x and the ratio of absolute values of x and y-directional velocities $|v_y|/|v_x|$ of ECs for 6 h while exposing the cells to the four different oxygen and flow conditions N_{stat}, $H3_{stat}$, N_{flow}, and $H3_{flow}$. Error bars show the standard deviation.

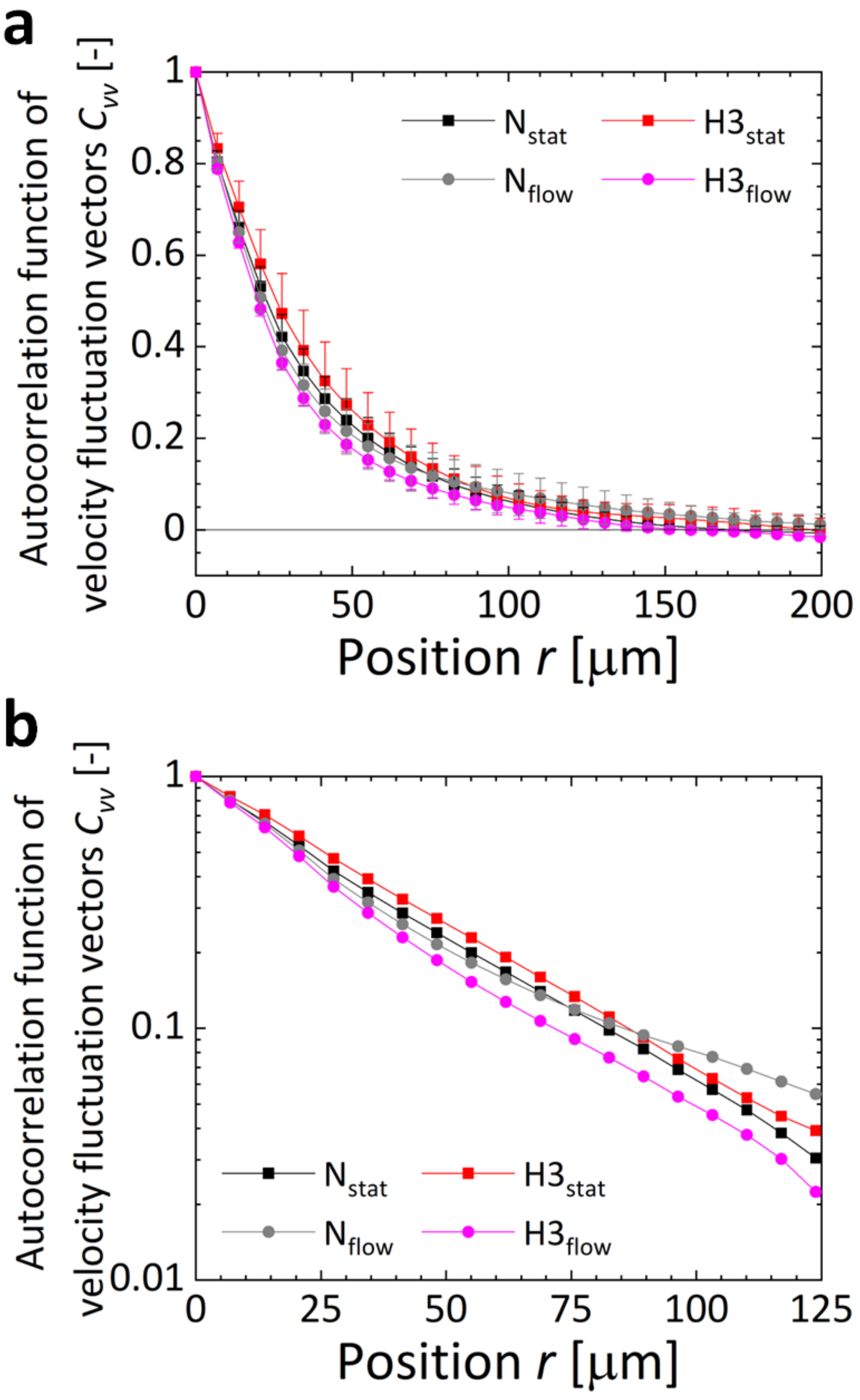

Fig. S10 Autocorrelation function of velocity fluctuation vectors C_{vv} of ECs in the monolayer under the conditions N_{stat}, $H3_{stat}$, N_{flow}, or $H3_{flow}$: (a) the profile on a linear scale and (b) the decay on a logarithmic scale. Error bars show the standard deviation.

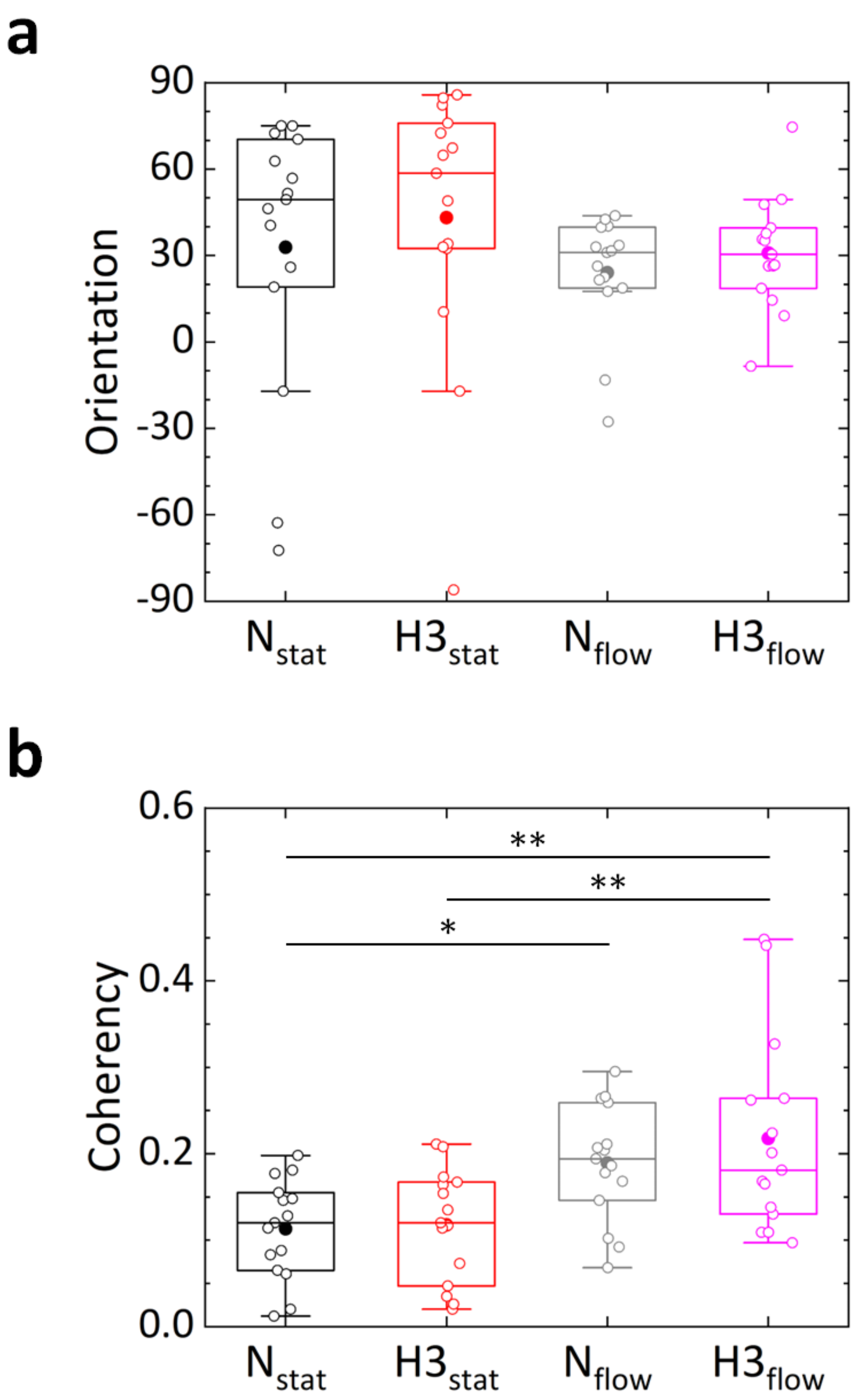

Fig. S11 Morphological changes of actin filaments in ECs in the monolayer after 6 h of exposure to the four different oxygen and flow conditions N_{stat}, $H3_{stat}$, N_{flow}, and $H3_{flow}$. Box-and-whisker plots of (a) orientation and (b) coherency. The upper and lower extremes represent the maximum and minimum values, the box plot represents quartiles, and the band and dot inside each box show the median and average value, respectively. The metric was measured with 15 images from three devices for each condition. Significant differences in the metrics at different oxygen and flow conditions were assessed by 2-way ANOVA followed by Tukey's post-hoc tests for multiple comparisons. $*P < 0.05$; $**P < 0.01$.

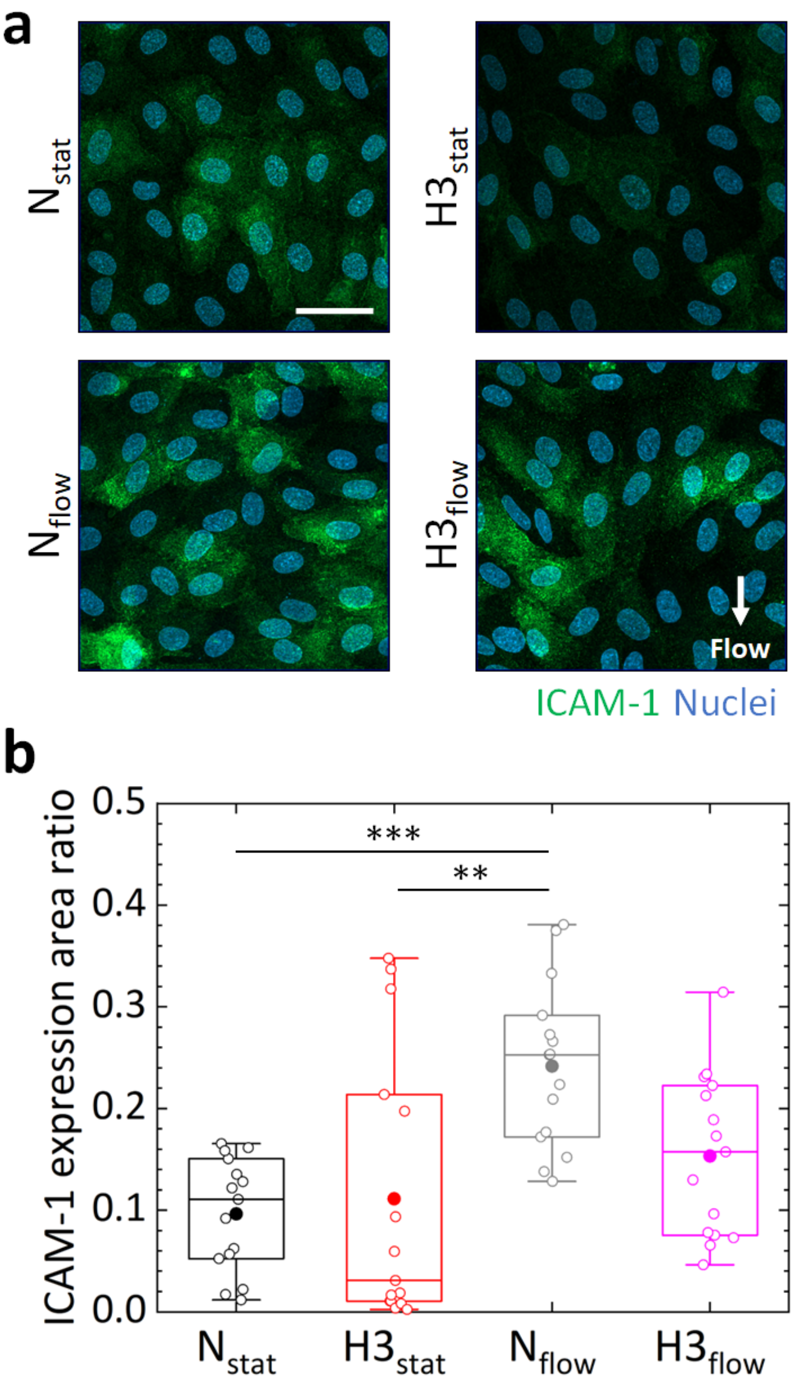

Fig. S12 ICAM-1 expression on ECs in the monolayer after 6 h of exposure to the four different oxygen and flow conditions N_{stat}, $H3_{stat}$, N_{flow}, and $H3_{flow}$. (a) Representative images of maximum intensity projections of confocal microscope images of ECs to the *xy*-plane. Scale bar shows 40 µm. (b) Box-and-whisker plots of the ICAM-1 expression area ratio over the imaging area with the raw data plotted. The upper and lower extremes represent the maximum and minimum values, the box plot represents quartiles, and the band and dot inside each box show the median and average value, respectively. The metric was measured with 15 images from three devices for each condition. Significant differences in the metrics at different oxygen and flow conditions were assessed by 2-way ANOVA followed by Tukey's post-hoc tests for multiple comparisons. $^{**}P < 0.01$; $^{***}P < 0.001$.

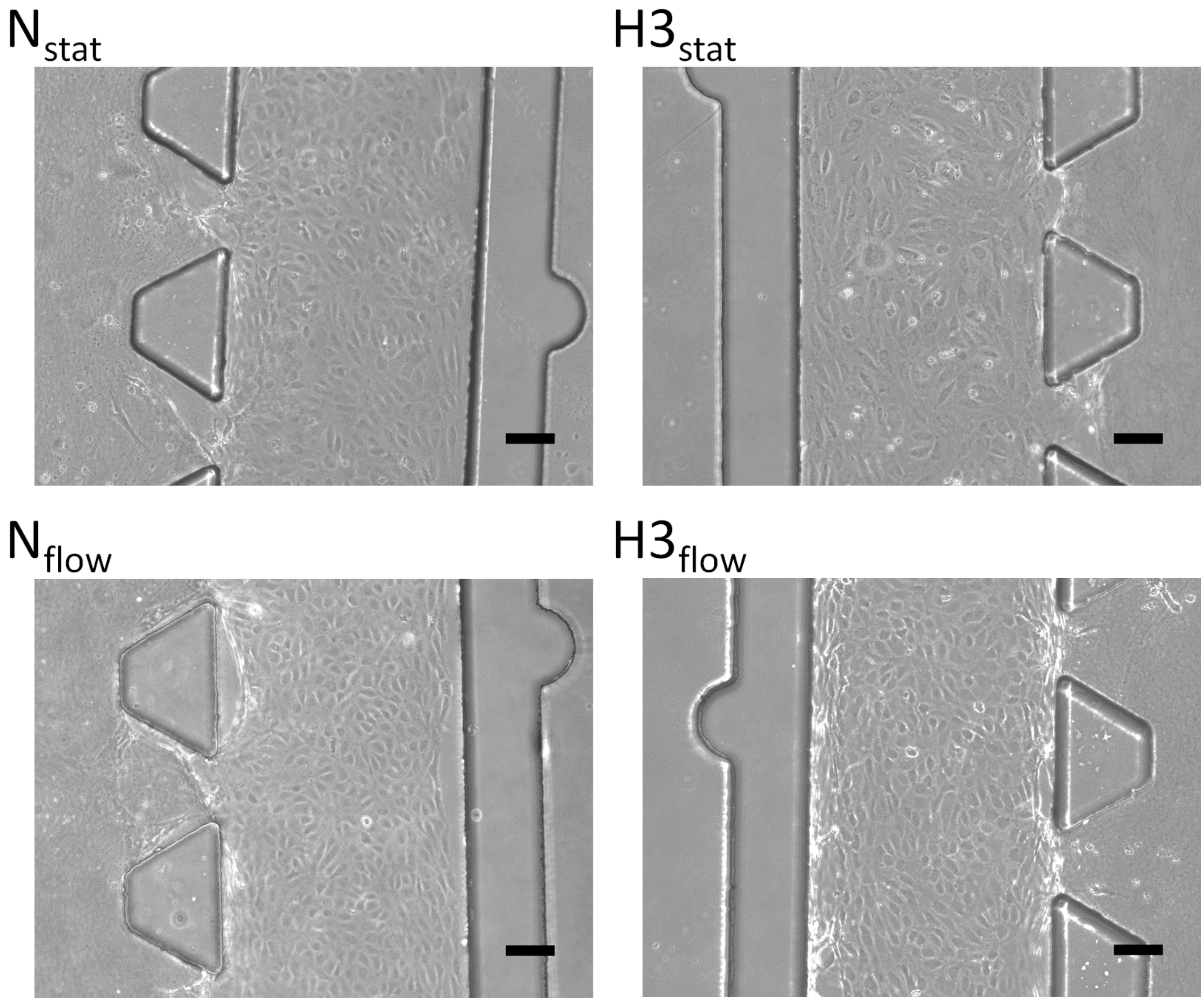

Video S1-S4 The collective cell migration of the HUVECs for 5 h at 3,600× speed under the conditions N_{stat}, $H3_{stat}$, N_{flow}, or $H3_{flow}$. Scale bar = 100 µm.
